# Observation of rescue behaviour in wild boar (*Sus scrofa*)

**DOI:** 10.1038/s41598-021-95682-4

**Published:** 2021-08-10

**Authors:** Michaela Masilkova, Miloš Ježek, Václav Silovský, Monika Faltusová, Jan Rohla, Tomáš Kušta, Hynek Burda

**Affiliations:** Department of Game Management and Wildlife Biology, Faculty of Forestry and Wood Sciences, Czech University of Life Sciences, Prague, Czech Republic

**Keywords:** Evolution, Zoology

## Abstract

Here, we provide unique photo documentation and observational evidence of rescue behaviour described for the first time in wild boar. Rescue behaviour represents an extreme form of prosocial behaviour that has so far only been demonstrated in a few species. It refers to a situation when one individual acts to help another individual that finds itself in a dangerous or stressful situation and it is considered by some authors as a complex form of empathy. We documented a case in which an adult female wild boar manipulated wooden logs securing the door mechanism of a cage trap and released two entrapped young wild boars. The whole rescue was fast and particular behaviours were complex and precisely targeted, suggesting profound prosocial tendencies and exceptional problem-solving capacities in wild boar. The rescue behaviour might have been motivated by empathy because the rescuer female exhibited piloerection, a sign of distress, indicating an empathetic emotional state matching or understanding the victims. We discuss this rescue behaviour in the light of possible underlying motivators, including empathy, learning and social facilitation.

## Introduction

Rescue behaviour in animals is a form of prosocial action when one individual (rescuer) frees the other individual (victim) that finds itself in a distressing or dangerous situation^1^. Rats freeing restrained cage mates and ants rescuing their colony members trapped in a nylon snare buried in sand represent concrete examples of this phenomenon^2,3^. Rescue behaviour differs from other forms of helping by its complex organization. To qualify as rescue behaviour, the behaviour must meet four requirements^1^. First, the victim must be in distress, finding itself in a situation imposing a physical threat such as injury or death. Second, the rescuer puts itself at risk by attempting to free the victim; the rescue attempt represents a potentially great cost to the rescuer and thus is considered as extreme form of prosocial behaviour. Third, the actions of the rescuer are adequate to the victim’s situation, even if the rescue attempt turns out to be unsuccessful. Finally, there is no immediate benefit for the rescuer in terms of food rewards, social contact, protection, or mating opportunities. Indirect benefits such as fitness benefits are, however, possible^1^.

Rescue behaviour is considered a form of targeted helping, along with other prosocial behaviours such as instrumental helping^4^. According to the Russian doll empathy model^5,6^, targeted helping, together with consolation, represent the most complex forms of empathy that require perspective taking and emotional state matching. Yamamoto (2017)^7^ suggested an alternative combination model of empathy consisting of three independent factors that might interact: matching with others, prosociality, and understanding of others. In this model, rescue behaviour is placed under factor prosociality and does not involve the emotional matching or understanding others. However, rescue behaviour does not have to involve the empathetic component at all and there might be other motivators behind it^8,9^. Underlying motivations of rescue behaviour are difficult to reliably study due to experimental design or because several motivators can act simultaneously. Most of the studies do not provide data on motivations and, thus, the discussion on the underlying mechanism of rescue behaviour is ongoing^8–12^.

Experiments with laboratory rodents emphasized the importance of experimental design to verify empirically the empathic motivators. In the first condition of the experiment of Ben-Ami Bartal et al. (2011)^3^, rats opened a front door of a restraint tube and released their trapped cage mates into the same chamber. In the second condition, rats opened a rear door of the restraint tube and released the trapped cage mates into a distal chamber. Thus, they continued helping their trapped cage mates even in situations when social contact was prevented. This led the authors to the conclusion that rats understood the state of distress of their cage mates and acted to alleviate it and, thus, that the rescue behaviour was empathy motivated. This conclusion was supported by a study in which rats opened a door and freed a distressed, soaked cage mate from a water tank^13^. Moreover, rats did not open the door for soaked cage mates that were not in distress. The follow-up studies replicated the rat helping paradigm in slightly different arrangement and challenged the interpretations of the original study. Silberberg et al. (2014)^14^ suggested social contact seeking as an alternative underlying mechanism because rats did not open the rear door to release the trapped cage mates into a distal chamber when the order of experimental conditions was reversed. Similarly, rats showed no preference between releasing a restrained individual or spending time with a not restrained individual^15^. Furthermore, a recent study on mice suggested that rescue behaviour might be motivated by general interest in the restraining apparatus^16^. Finally, Hachiga et al. (2020)^12^ proved that the stay in the restraint tube was not stressful for the trapped rats but rather rewarding. Therefore, the design of experimental paradigm, measuring physiological or emotional responses to restraint, and the succession of rescuer actions are crucial in disentangling the motivators of rescue behaviour^1,8,16–18^.

Whether driven by empathy or not, some researchers suggest that rescue behaviour might also be present in other taxa^1,19^. However, because of the rare occasions where this phenomenon is documented, only a few studies on a handful of species are available to support this claim (see Table 1). Rare experimental evidence includes ants freeing their entrapped conspecifics^2,11,20^ and dogs releasing their distressed owners^21^. In other cases, we have to rely on observational evidence and case reports. Nonetheless, observational reports can be informative not only about the various taxa in which apparent rescue behaviour was observed, but also about various forms of rescue behaviour. For example, a male white-faced capuchin monkey was observed intervening during aggression towards a mother and her infant that escaped into a river after an attack by males from a neighbouring group, thus preventing her death^22^. Other observational evidence includes chimpanzees removing poacher´s snares from conspecifics limbs^23^ or elephants removing tranquilizing darts from the body of their conspecific^24^ that would otherwise have resulted in capture by humans. A recent observational report also documented the first evidence of rescue behaviour in birds. Seychelles warblers were reported to remove the sticky seeds of the “bird-catcher tree”, that prevent flying and can result in death, from the feathers of their group members^19^.

**Table 1:** Examples of studies documenting rescue behaviour in animals.

Common name	Scientific name	Condition	Context of rescue behaviour	Reference
***Carnivora***				
Dog	*Canis lupus familiaris*	E	Opening door to release owner trapped in a box	^21,25^
Banded mongoose	*Mungos mungo*	O	Lunging at an eagle to release captured pack member	^26^
***Cetartiodactyla***				
Humpback whale	*Megaptera novaeangliae*	O	Interfering with killer whale to release attacked conspecifics	^27^
Wild boar	*Sus scrofa*	O	Opening door to release group members trapped in a cage	this study
***Primates***				
Black-tufted-ear marmoset	*Callithrix penicillata*	O	Cooperative attack on a snake to rescue captured group member	^28^
White-faced capuchin	*Cebus capucinus*	O	Intervening in deadly attack of female and her infant by another individual	^22^
		O	Cooperative attack on a snake to release captured group member	^29^
	*Cebus imitator*	O	Cooperative attack on a snake to release captured group member	^30^
Grey mouse lemur	*Microcebus murinus*	O	Cooperative attack on a snake to release captured group member	^31^
Bonobo	*Pan paniscus*	O	Removing snare to release captured conspecific	^32^
Chimpanzee	*Pan troglodytes*	O	Removing snare to release captured conspecific	^23^
		O	Cooperative attack on a leopard to rescue group member	^33^
Bornean orangutan	*Pongo pygmaeus*	O	Protecting a female attacked by other individuals	^34^
Coquerel´s sifaka	*Propithecus coquereli*	O	Cooperative attack on a snake to release captured group member	^35^
Moustached tamarin	*Saguinus mystax*	O	Cooperative attack on a snake to release captured group member	^36^
***Proboscidae***				
African elephant	*Loxodonta africana*	O	Removing foreign objects (tranquilizing darts, spears) from body of conspecific	^24^
		O	Intervening in attack on conspecifics by other individuals	^24^
***Rodents***				
Rat	*Rattus norvegicus*	E	Opening door to release a cage mate entrapped in a restrainer	^3,14,15,17,18,37–39^
		E	Opening door to allow soaked conspecific into dry area	^10,13^
Mouse	*Mus musculus*	E	Opening lid to release a cage mate entrapped in a tube	^16,40^
***Birds***				
Seychelles warbler	*Acrocephalus sechellensis*	O	Removing sticky seeds from feathers of an entangled group member	^19^
***Invertebrates***				
	*Cataglyphis cursor, C. floricola, Lasius grandis*	E	Removing sand, pulling a limb and biting in snare to release a nest mate ensnared by a nylon snare and buried in sand	^2,11,20^
	*Formica sanguinea, F. cinerea, F. fusca*	E, O	Removing sand and pulling a limb of nest mate captured by an ant lion larva	^41^
	*Veromessor pergandei*	O	Removing spiderwebs to release entrapped nest mates	^42^

E = experiment, O = observation. The cases involving rescue from predators are often termed as examples of cooperative self-defence against predators^19^.

**Figure 1:**
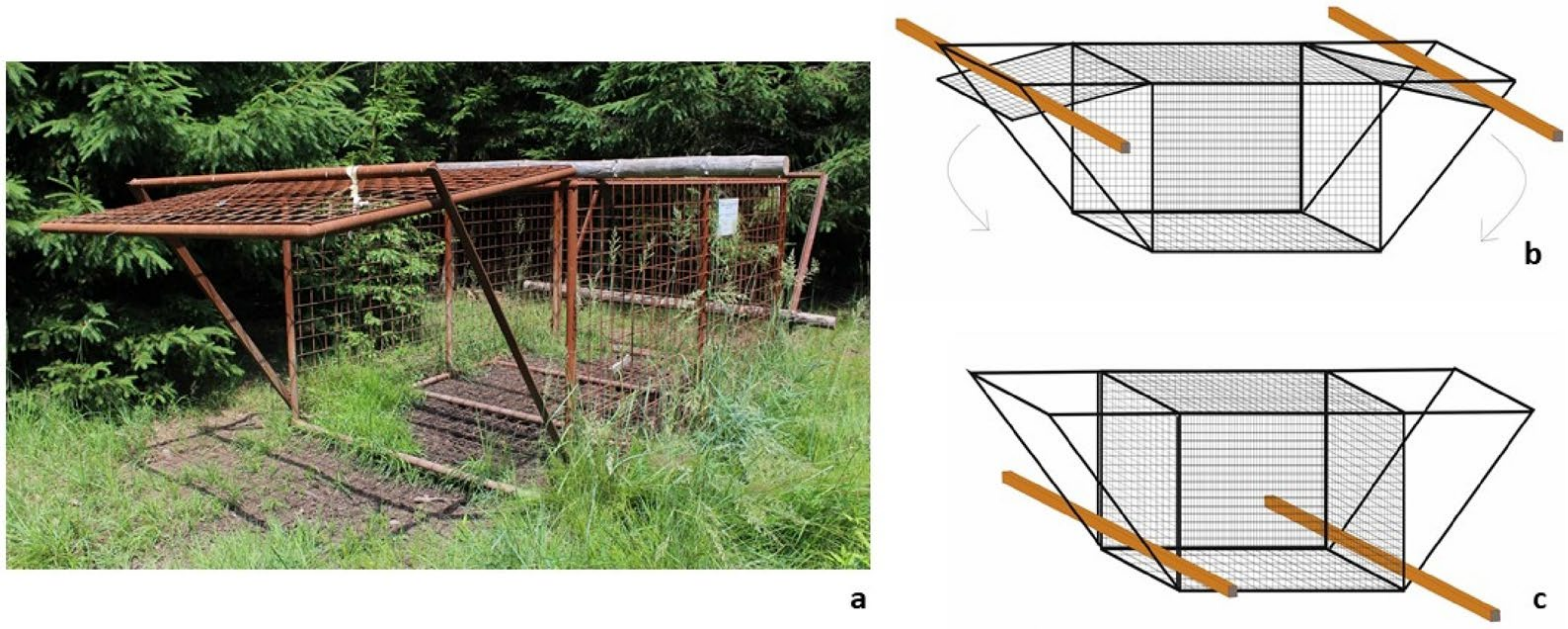
A box trap used for trapping wild boar at the study location (**a**). The open tipping door is secured by a wire. One end of the wire is attached to the door and the other end is hooked to the bottom of cage. The door is closed by manipulating the hook of the wire from inside the trap, usually during rooting. For a detailed photo of trigger mechanism, see Supplementary electronic material, SEM (Fig S1). Triggering the mechanism closes the door and releases the logs which secure the doors from the outside (**b**,**c**).

Here, we report the first observational evidence and photo documentation of a potential case of rescue behaviour in wild boar (*Sus scrofa*), a species which, due to its nocturnal activity, is rarely studied in the wild in terms of social cognition. In the observed case, an adult female freed two juvenile boars from a cage trap (Fig. 1). The incident was recorded by a camera trap set to take a photo every two minutes and installed for monitoring the visitation of a trap baited with corn. The trapping is employed to individually mark wild boars that are part of a study on movement ecology and African Swine Fever prevention measures. We discuss this case in light of the four requirements of rescue behaviour and possible underlying motivators^1^.

## Results

The incident occurred on the night from 28 to 29 January 2020 and was captured in 93 photos (available in high resolution in SEM). Two boars were entrapped together for 2 h and 35 min. The other boars arrived at the trapping site after 2 h and 6 min of the two being trapped, and the whole apparent rescue event from the first contact of the opening mechanism to the last available photo took 29 min, with the first successful removal of a log after 6 min.

From 21:00 to 21:04 h, the brief presence of four wild boars was noted near the cage trap. Boars were moving at the rear and side part of the trap for a total time of 4 min and then left the trapping site.

**Figure 2﻿:**
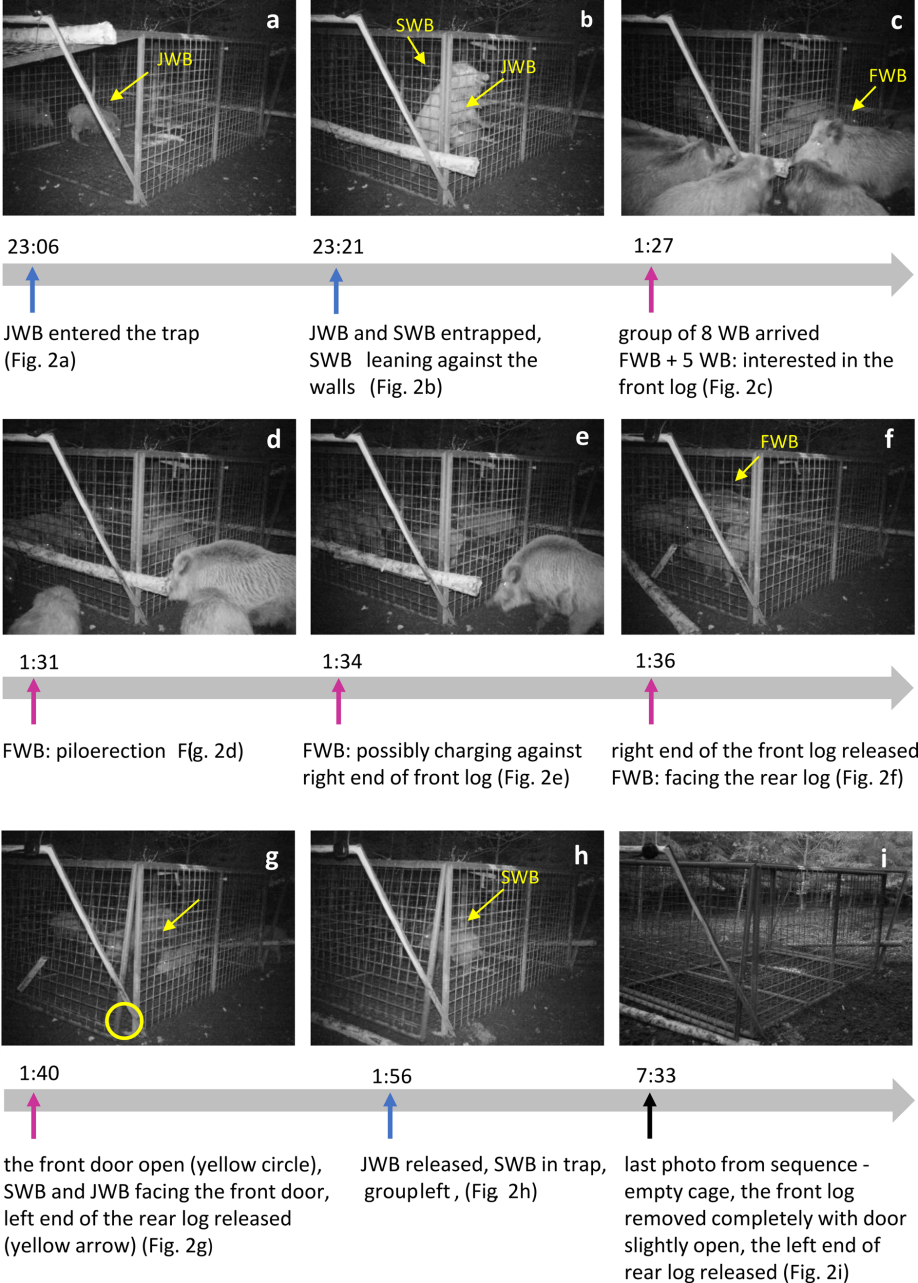
Timeline and photos documenting the potential rescue event by wild boar. Victims: SWB = subadult wild boar, JWB = juvenile wild boar; rescuer: FWB = female wild boar. The behaviour of the potential rescuer is indicated by pink arrows and the behaviour of victims by blue arrows in the timeline.

The camera trap was triggered again at 23:02 h, which is considered the beginning of the apparent rescue event. The detailed timeline and photos of the event are depicted in Fig. 2. At 23:02, two wild boars (WB) of unknown sex appeared outside of the trap: a juvenile (JWB) — a smaller individual with visible remnants of piglet stripes — and a subadult (SWB) — a somewhat bigger but not fully grown individual. The two WB were moving along the left side of the trap and, at 23:06, JWB entered the trap (Fig. 2a). JWB was observed feeding on the corn in the trap for 15 min while SWB remained outside. At 23:21, the SWB entered the trap and triggered the door mechanism entrapping both JWB and SWB inside the trap (for the door mechanism, see Fig. 1 and Fig. S1 in SEM). From that time onwards, entrapped SWB and JWB were observed running, actively moving in the cage, and charging/leaning against the walls of the cage (Fig. 2b), exhibiting signs of distress. At 23:58, two WB were noted passing in the distance by the trap.

At 01:27, a group of WB (minimum eight) arrived at the trapping site, including one fully grown female (FWB) and seven subadults of unknown sex that were of a similar size as SWB. The FWB and five subadults immediately showed interest in the log securing the front door, looking at it and touching it with their snouts (Fig. 2c). Subsequently, the group dispersed around the trap, staying a maximum of 2 m away from it. At 01:31, the FWB faced the trap with the mane visibly erected, showing clear signs of piloerection (Fig. 2d). At 01:34, the FWB apparently charged against the front log with her head in a posture with bended back and erected mane (Fig. 2e). At 01:36, the right end of the front log was released while the left end stayed in its place. The FWB subsequently moved to the rear log which was securing the rear door and faced the side of the door (Fig. 2f). From this photo, it is not evident if the FWB manipulated the log, but in the next photo at 01:38 this part of the log was released (see SEM), suggesting that the FWB released the left end of rear log. Although it is evident from the whole photo sequence that WB had been manipulating the rear log based on its position, the right end of the rear log stayed securely blocking the opening of the rear door until the end of the whole event. At 01:40, the front log was removed completely and front door was slightly open. The door can only be opened by boar from inside pushing into it. Although in Fig. 2g the SWB and JWB were depicted facing the door and potentially charging against it, they stayed entrapped for another 15 min. At 01:49, the FWB was depicted facing the side of front door potentially charging against it in the same manner as before (see SEM). At 01:54, the front log appeared again in the photo, suggesting that WB had been moving it.

The last photo was taken at 01:56 and, because there was no further motion in front of camera trap and thus no other photographs available from that night, we consider the time 01:56 as the end of the event. The last photo documented the SWB inside the trap with the front door slightly open, completely removed front log, and removed left end of the rear log (Fig. 2h). The JWB had escaped through the gap in the door and left with the rest of the group from the sight of the camera trap. After 01:56, the SWB must have escaped through the open door because there was no other movement that would have triggered the camera trap. The cage box was found empty in the morning (Fig. 2i).

## Discussion

We observed a potential rescue event in which an adult female wild boar was documented in a series of photographs to act to free two wild boars that were caught in a box trap. The whole event lasted 29 min and involved several well-aimed attempts to remove the logs blocking the doors of the trap. The log was successfully removed after only 6 min. From the photo sequence, it appears that the female started her rescue immediately after she arrived with her group at the trapping site and the group left immediately after the entrapped boars were released.

The observed behaviour complies with the requirements of Nowbahari and Hollis (2010)^1^ to be qualified as rescue behaviour. The first requirement states that the victim is distressed and endangered by physical injury or death. Entrapped wild boars exhibited clear behavioural signs of distress, running in the cage and charging into the walls, possibly trying to escape, which resembles the signs of distress observed during trapping in other studies^43,44^. Wild boar are commonly trapped for management or research purposes. In the former case, they are shot by a rifle in the trap. In the latter case, they are handled to collect data or to be marked and released^43,45^. In either case, an increasing number of studies confirm that entrapment is extremely stressful for the animals involved, which can suffer from behavioural or physiological distress, immediate physical injuries or pathologies (e.g., capture myopathy or hyperthermia) sometimes resulting in death^43–45^. Without a video record, we are not able to say if any distress vocalizations (e.g. grunts, squeals or grunt-squeals) were involved^46^. However, taken together, we can state that the entrapped boars found themselves in a stressful and potentially dangerous situation that could cause them physical harm^44,45^.

According to the second requirement of rescue behaviour, the rescuer puts itself at risk by its rescue actions^1^. Although it is not possible that the rescuer female could have been entrapped, by removing the log and charging into the wire mesh, she risked physical injury and, by spending time at a potentially dangerous place, she risked being harmed herself. This is similar to ant research in which the rescuer ant risked being buried by sand or caught by a predator but could not be ensnared any more^2^. The third requirement proposes that the behaviour of the rescuer is adequate to the victim’s circumstances. In the case of the wild boar, the rescuer female acted immediately to remove the log. When she was unsuccessful with the first log, she tried to remove the second log. After removing the logs, she charged from the side of the door. Her behaviour thus helped the entrapped conspecifics that were able to open the door from inside. According to the fourth requirement, rescue behaviour does not bring any direct benefit to the rescuer, which was met in our case. Even though the trap was baited with attractive food, the group left the trapping site immediately after opening the cage, leaving leftovers of corn in the trap, suggesting that the rescue action was not food motivated. The absence of further photos from that night proves that the group did not return to the trapping site. Indirect benefits, in terms of kin selection or reciprocal altruism, are possible^1,47^.

**Figure 3:**
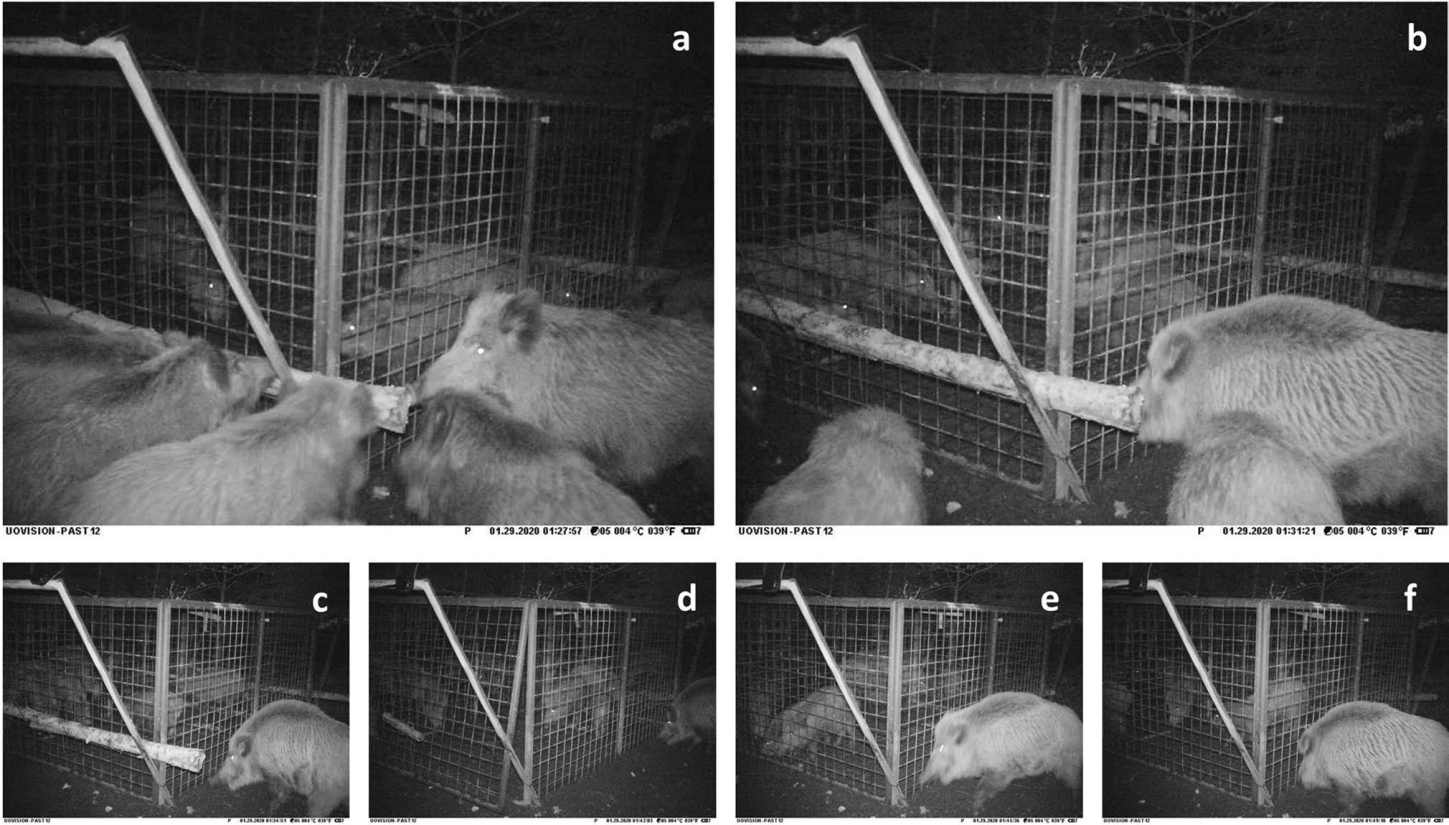
The piloerection of adult female wild boar during the rescue behaviour. See (**a**) and (**b**–**f**) for comparison of non-piloerected and piloerected fur.

There are several contributing factors that facilitates rescue behaviour. The sex of the rescuer is one of them. Based on the photo series and sequence of her actions (e.g. Figure 2d-f, Fig. 3), we assumed that the rescuer was in this case the adult female. Similarly, in the rat restraining paradigm, a greater proportion of females than males released the restrained cage mate^3^. Sex, however, didn’t affect the rescue behaviour in dogs^21^. Although the sex of the rescuer as a factor of rescue behaviour has not been systematically studied, the effect of sex might be species specific and related to the social system. From Fig. 2c it also appears that other individuals showed interest in the log securing the door. Thus, it is possible that they could have also been involved in rescuing the entrapped boars and that the rescue was, in fact, cooperative. Cooperative rescue behaviour would not be surprising due to frequent cooperative interactions and close social bonds in boar^48,49^. We are, however, not able to prove this without detailed evidence. Thus, we consider the female as the sole rescuer in further discussion. Another contributing factor to the rescue behaviour is the relationship of rescuer and victim. Because of the relatively small body size of the entrapped individuals, it is presumed that the female might have been their mother and that the boars where part of one group^49^. The relatedness of the boars involved in this incident is, however, unknown. Nevertheless, according to Nowbahari and Hollis (2010)^1^, rescuing kin also qualifies as rescue behaviour. In fact, in most of the examples of rescue behaviour, the victim was somebody familiar to the rescuer, such as a cage mate^3^, pet owner^21,25^ or group member^26,28,31^. In a few cases, the rescue behaviour was directed to unfamiliar conspecifics or even to individuals of different species^27^. Familiarity, therefore, may facilitate rescue behaviour due to benefits connected to kin selection or reciprocal altruism^1,47^.

We are thus convinced that we provide evidence of rescue behaviour observed for the first time in wild boar and in Suidae. Although Artiodactyla are phylogenetically related to Cetaceans which have been demonstrated to have complex social cognitive skills, including rescue behaviour^27^, rescue behaviour has not been observed in any other Artiodactyla family. Documenting rescue behaviour in wild boar is, however, not surprising due to their complex cognitive skills and social relationships^49,50^. Our observation of rescue behaviour was very similar to the experimental studies on ants, rats and dogs, where one individual freed other individuals that were restrained or entrapped in either an experimental box or by a snare^2,3,21^.

The question, however, remains whether this case of rescue behaviour was motivated by empathy^9,51^. Although we do not have physiological or detailed behavioural data to confirm whether the rescuer understood or shared the emotions of the victims (i.e., evidence of cognitive perspective taking or emotional state matching^52^), we cannot rule out this possibility either. As was discussed above, trapping represents a highly stressful situation for the entrapped wild boars that exhibited signs of distress. The rescuer female had an erected mane or arched back^53^, both being displays of intimidating or threatening behaviours^54^ and acute stress response (i.e., "flight or fight")^55^. The fact that she exhibited signs of piloerection in more than half of the photos in which she was present (see Fig. 3 for details) may imply possible physiological arousal of the female when watching others in distress and potentially even matching emotional state. She also continuously stayed in proximity to the cage and often looked at the victims. Thus, it is possible that the rescuer female either perceived the situation as dangerous (cognitive perspective taking) or perceived the emotional state (emotional state matching) of the entrapped boars and acted to alleviate it. Therefore, the rescue behaviour in wild boar might have been empathy motivated.

In fact, previous studies have confirmed the existence of empathy in domestic pigs demonstrating emotional contagion^56,57^ in various contexts^58,59^ . Emotional contagion was also documented in the situation of restraint, when a naive pig showed signs of stress after watching a conspecific in a stressful situation^59^. Emotional contagion is considered as a basic block of empathy according to the Russian doll model^5,6^ and as one of the factors of empathy in the combination model of empathy^7^. Another study proved cognitive perspective taking in domestic pigs^60^. The presence of empathy in wild boar is expected. Wild boars live in dynamic matrilineal societies composed of females and their offspring of several generations, with fission–fusion patterns and complex social relationships^49,61^. The group units of wild boar are cohesive with strong social bonds^62^ and frequent social behaviour, including cooperation^48^ and other forms of prosocial behaviour (e.g., alloparental care^63^), which are conditions favouring the emergence of empathy^64^. Therefore, it seems that empathetic behaviours, in various forms, are present in Suidae and our report of rescue behaviour might represent additional evidence. In the case that the emotional state matching (exhibited e.g. as piloerection) was indeed involved as discussed above, the rescue behaviour represents the most complex form of empathy according to the Russian doll model^5,6^ rather than prosociality factor in the combination model of empathy^7^.

Prosocial behaviour, however, does not have to be motivated only by empathy^8^. Other alternative underlying mechanisms, such as social contact seeking^14^, a selfish act to terminate the signals of stress^8,65^, curiosity^37^, and an opening of the trap by accident, have been proposed. We can, however, rule out most of these hypotheses. First, the rescuer female was part of a large group which could easily provide social contact if desired. Second, the female could have just ignored the stress of the entrapped animals and left the trapping site even though observing distressed conspecific could induce personal distress in the observer itself^65^. Third, the female did not explore the box trap. Moreover, the wild boars in this area are familiar with box and corral traps as they are common methods of game management and wildlife biology research. However, we cannot rule out curiosity completely based only on the photographic evidence. Fourth, the rescue behaviour represents a rather complex sequence of actions (attempting to release both logs and charging into doors, both requiring substantial strength) rather than coincidentally opening the mechanism caused by the proximity of the rescuer.

It is, however, possible that the rescuer female had previous experience with opening the cage doors. In two separate instances preceding this case report, we had received an alert that the animals were entrapped, but when we arrived at the cage, it was empty. Unfortunately, we do not have any photo material to document what happened and which species or individuals were involved. Learning and previous experience was suggested as a potential motivator of releasing behaviour in rats^37^ and dogs^21^. In rats, the opening latency decreased across trials, indicating a learning curve^37^. Social facilitation learning is another alternative explanation in which the presence of conspecifics could have influenced the behaviour in others^37^. For example, the rescue behaviour of the female could have been triggered or facilitated by the presence of other boars as well as influenced to be more efficient or fast. Both learning and social facilitation could have potentially facilitated the rescue behaviour in wild boar. However, we do not have empirical evidence for this statement. These motivators are not mutually exclusive and several of them might have acted together. Finally, without further detailed exploration of underlying mechanisms, the motivator of rescue behaviour in wild boar remains unclear. Future empirical studies should focus especially on empathy, learning and social facilitation learning as potential motivators of rescue behaviour in wild boar or pigs to disentangle this problem.

Our photo documentation of this event is, however, not without flaws. Setting a camera trap to video regime or photo regime with a shorter gap between photos could have helped to disentangle the precise actions of the rescuer, possible involvement of other group member or signs of distress of the victims as well as the rescuer. Nevertheless, our observation represents rare and solid evidence of spontaneous complex rescue behaviour in wild boar. Observational evidence and case reports of biologically interesting phenomena represent a valuable contribution for understanding animal behaviour or cognition. In some cases, observational evidence is the only means to study certain rare behaviours or elusive species such as wild boar. Furthermore, case reports can facilitate further research and can help design experimental studies^66^. With our observational evidence of rescue behaviour in wild boar, we hope to facilitate the research of empathetic and cognitive abilities of wild boar, especially the motivators of rescue behaviour and the possible role of familiarity, sex and stress clues.

## Methods

This event was observed in Voděradské Bučiny National Nature Reserve, located east of Prague in the Czech Republic. The reserve covers 684 ha and is composed of mixed deciduous forest with beech as the predominant tree species. Wild boar are a common species in the reserve and the hunting bag varies from 5 to 15 individuals per km^2^ per year. The wild boar in this locality are subjects of intensive and long-term research of African Swine Fever prevention measures and movement ecology (funding No. QK1910462. financed by Ministry of Agriculture of the Czech Republic). The adult wild boars are regularly trapped using box cages or corral traps, which are monitored remotely by camera traps, and subsequently immobilized by experienced wildlife researchers. During handling, which takes approximately 20 min, physical measurements and tissue samples are taken. The trapped individual is marked with a plastic ear tag and fitted with a collar containing a GPS device and accelerometer (for details see ^67^).

Wild boars involved in the case of rescue behaviour were trapped in a box trap (3 × 2 × 2 m) made of steel wire mesh (mesh: 8 × 8 cm) (Fig. 1). The traps are baited with corn on a regular basis. The box trap has an open door allowing boars to enter the trap. The box trap is triggered by an individual releasing the door trigger mechanism on the opposite side of trap (see SEM Fig. S1). The whole incident was recorded by a camera trap (UOVision UM 595-2G with an effective detection distance of 12–15 m) attached to a tree at a height of 1.1 m and at a distance of 3 m from the cage trap, aiming at the open cage door. The camera covered the interior of the cage box and a perimeter of > 1 m around the cage trap. The camera trap was set to be launched by a movement, taking a picture every 2 min.

The wild boar trapping was implemented in accordance with the guidelines of the Ministry of the Environment of the Czech Republic. The trapping and handling protocol was approved by the ethics committee of the Ministry of the Environment of the Czech Republic. The study was carried out in compliance with the recommendations of ARRIVE guidelines^68^.

### Ethical approval

The wild boar trapping was realized in accordance with the decision of the ethics committee of the Ministry of the Environment of the Czech Republic number MZP/2019/630/361.

## Supplementary Information

Supplementary Information.
