# Supplementary figures and images for: Observation of rescue behaviour in wild boar (Sus scrofa)
Source: Sci Rep. 2021 Aug 10;11:16217. doi: 10.1038/s41598-021-95682-4 (PMC8355341; doi:10.1038/s41598-021-95682-4)

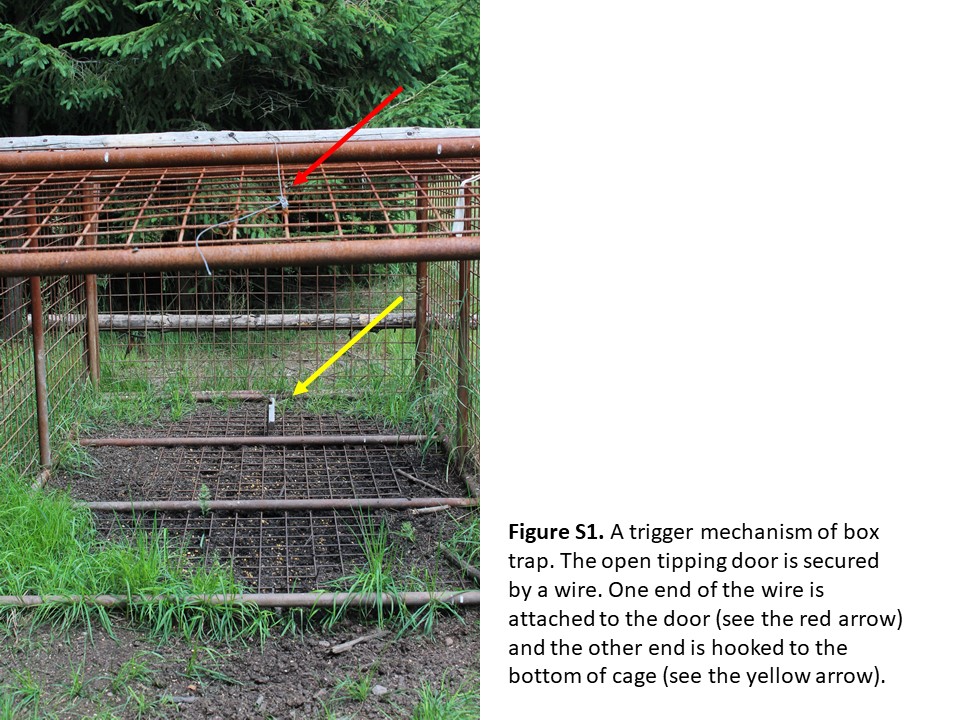

Supplement: Supplementary file 1 — Supplementary Information. [file 41598_2021_95682_MOESM1_ESM.zip › SEM_photos complete/Fig S1 trigger mechanism.jpg]

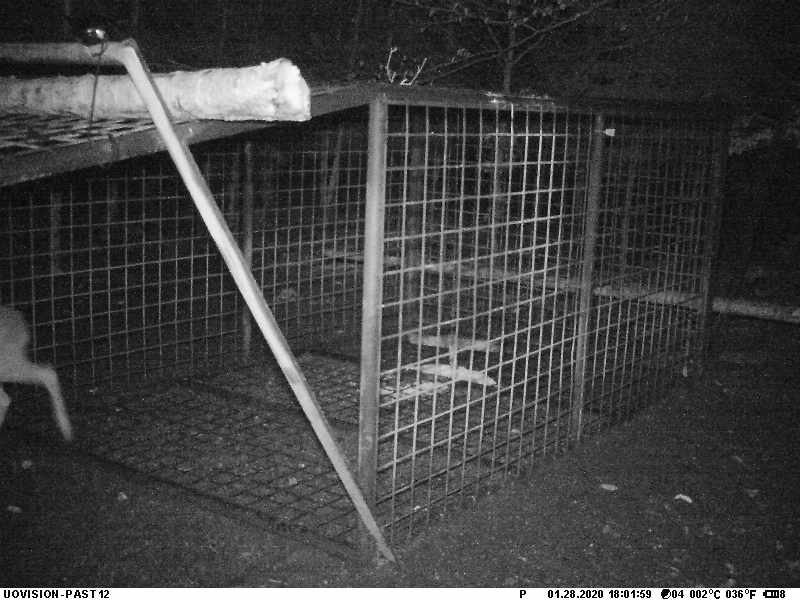

Supplement: Supplementary file 1 — Supplementary Information. [file 41598_2021_95682_MOESM1_ESM.zip › SEM_photos complete/PIC0688.jpg]

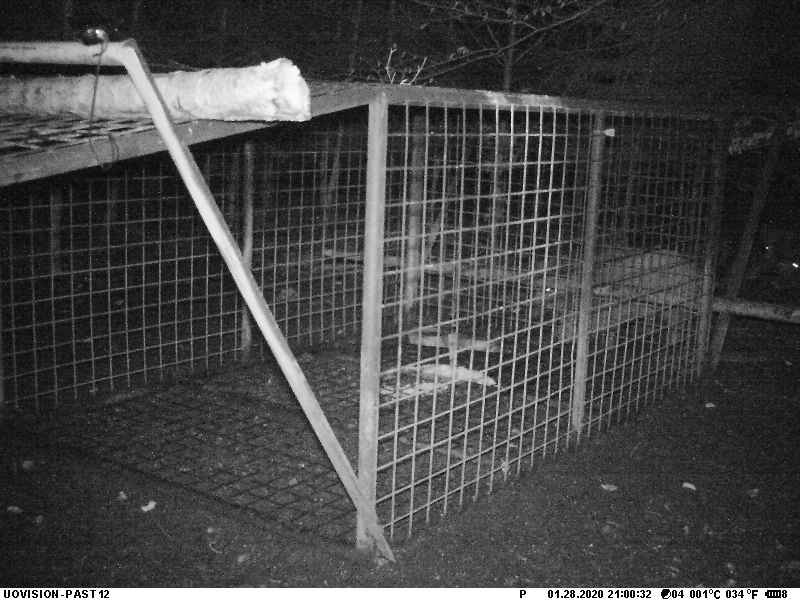

Supplement: Supplementary file 1 — Supplementary Information. [file 41598_2021_95682_MOESM1_ESM.zip › SEM_photos complete/PIC0689.jpg]

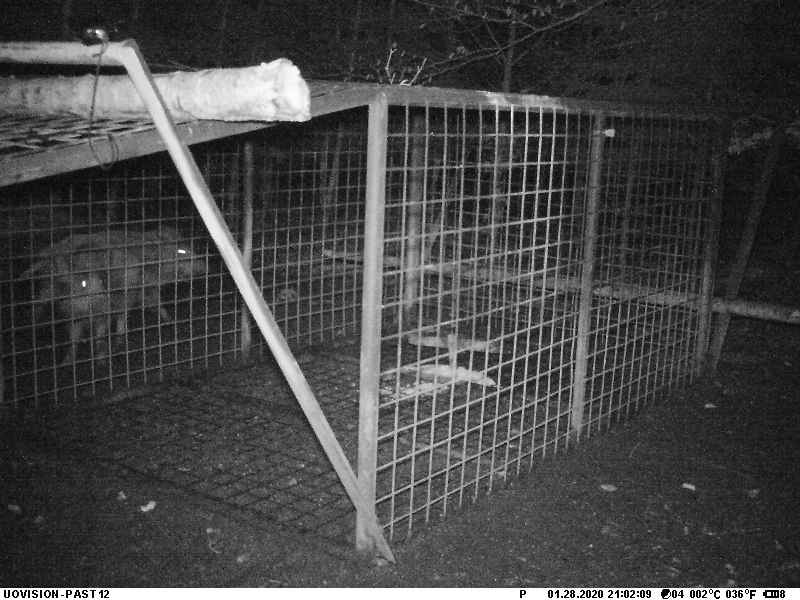

Supplement: Supplementary file 1 — Supplementary Information. [file 41598_2021_95682_MOESM1_ESM.zip › SEM_photos complete/PIC0690.jpg]

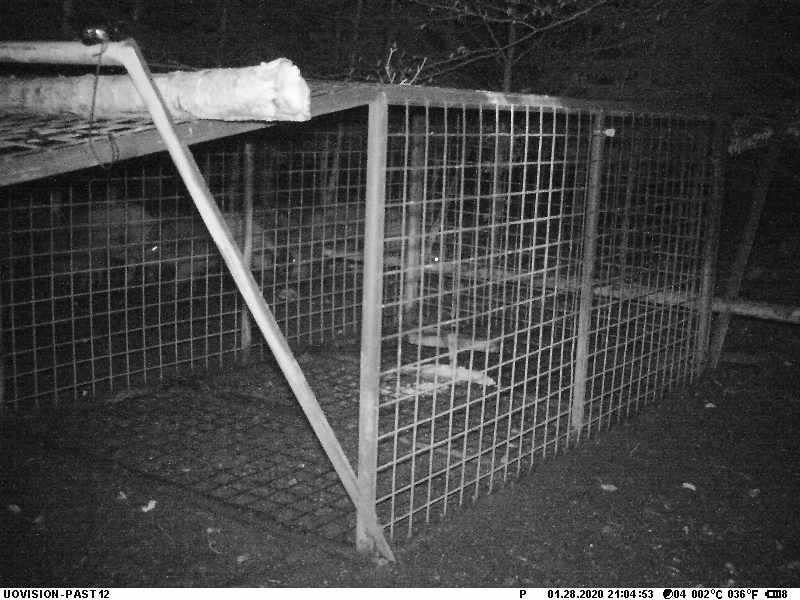

Supplement: Supplementary file 1 — Supplementary Information. [file 41598_2021_95682_MOESM1_ESM.zip › SEM_photos complete/PIC0691.jpg]

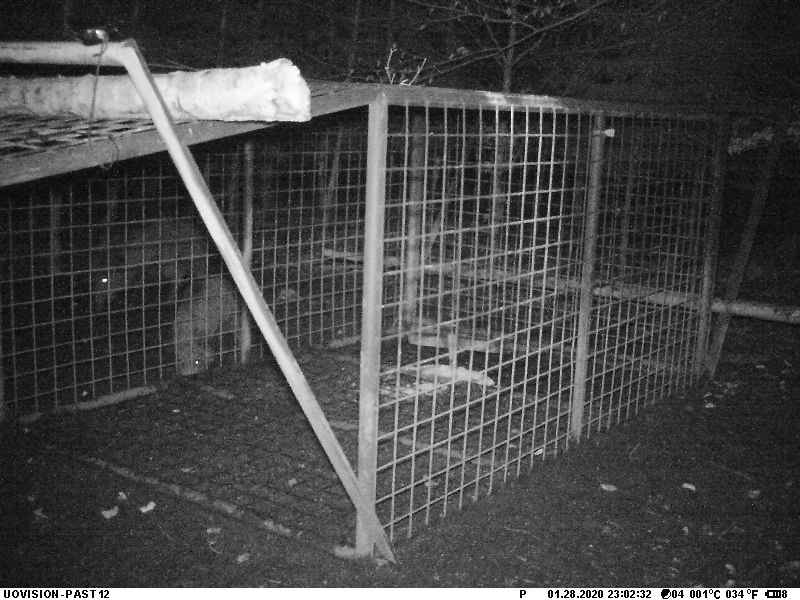

Supplement: Supplementary file 1 — Supplementary Information. [file 41598_2021_95682_MOESM1_ESM.zip › SEM_photos complete/PIC0692.jpg]

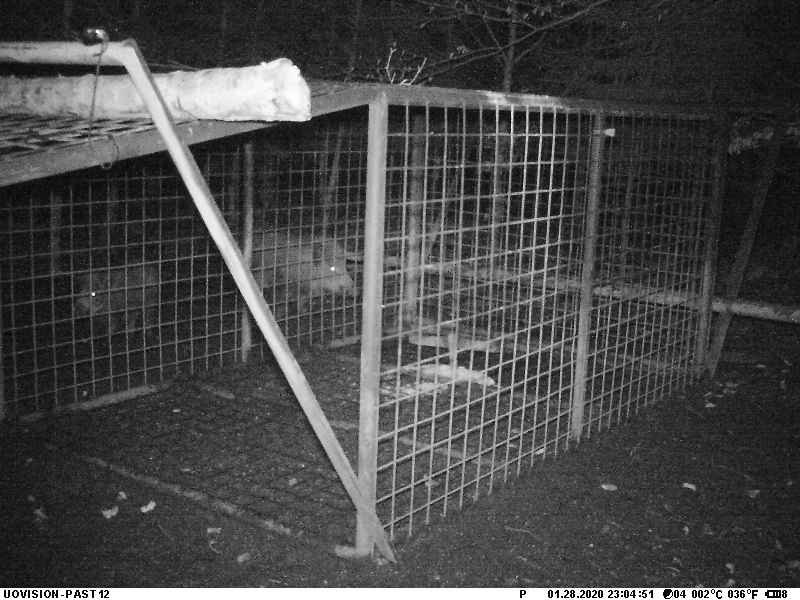

Supplement: Supplementary file 1 — Supplementary Information. [file 41598_2021_95682_MOESM1_ESM.zip › SEM_photos complete/PIC0693.jpg]

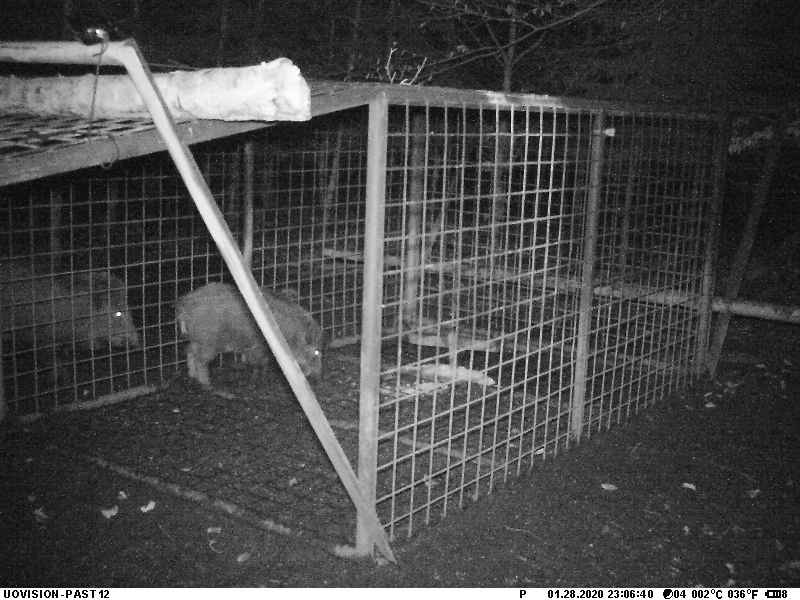

Supplement: Supplementary file 1 — Supplementary Information. [file 41598_2021_95682_MOESM1_ESM.zip › SEM_photos complete/PIC0694 (1).jpg]

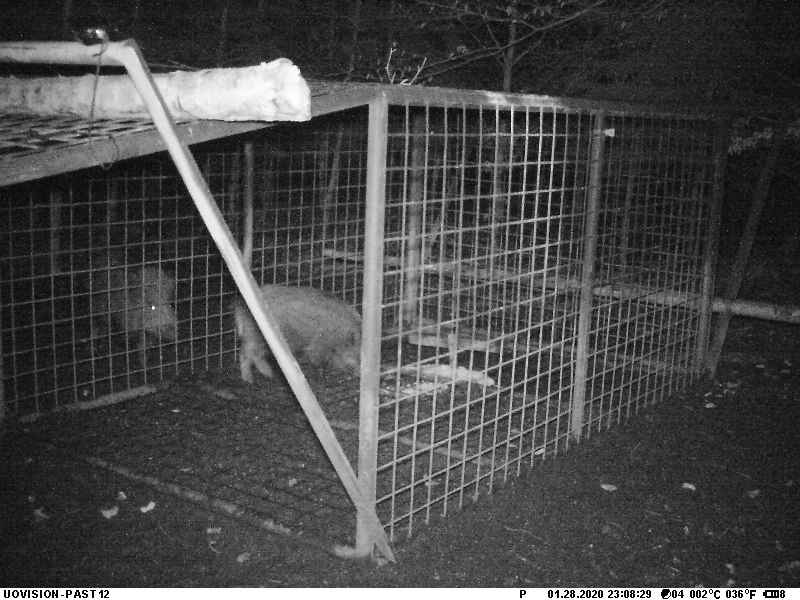

Supplement: Supplementary file 1 — Supplementary Information. [file 41598_2021_95682_MOESM1_ESM.zip › SEM_photos complete/PIC0695 (1).jpg]

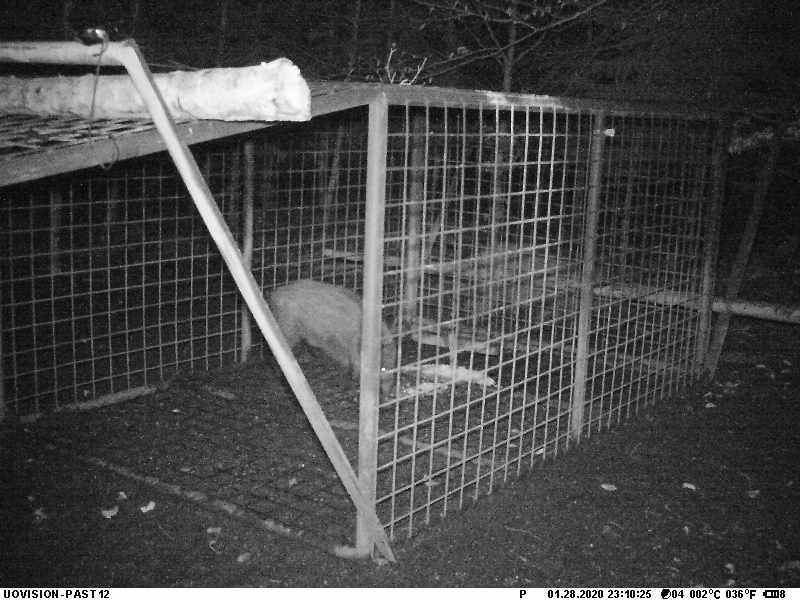

Supplement: Supplementary file 1 — Supplementary Information. [file 41598_2021_95682_MOESM1_ESM.zip › SEM_photos complete/PIC0696 (1).jpg]

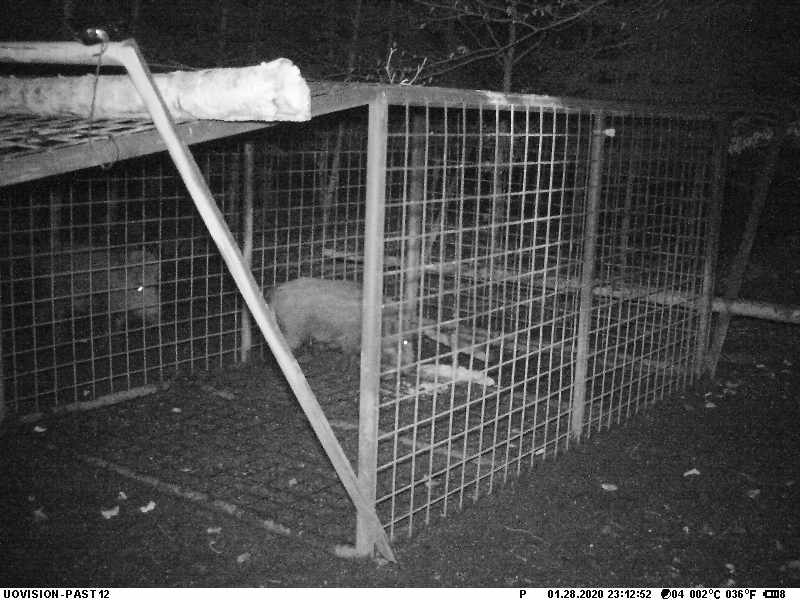

Supplement: Supplementary file 1 — Supplementary Information. [file 41598_2021_95682_MOESM1_ESM.zip › SEM_photos complete/PIC0697 (1).jpg]

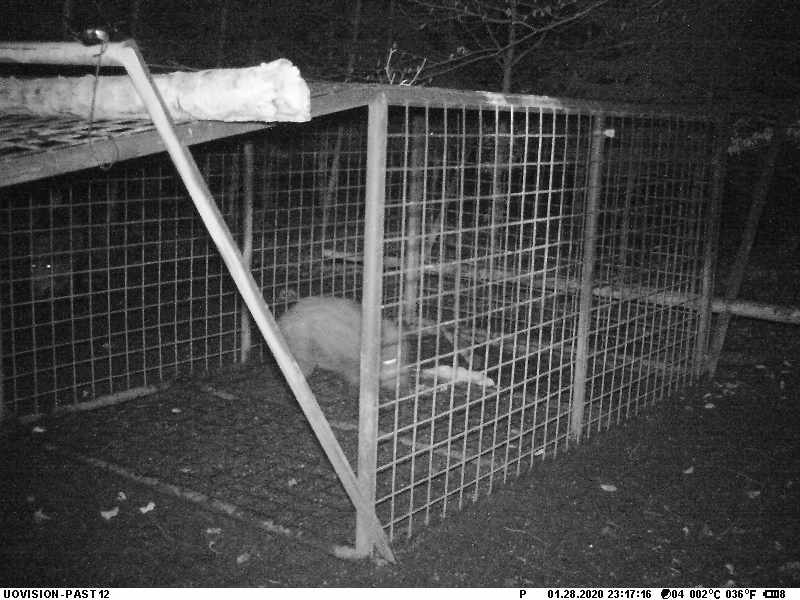

Supplement: Supplementary file 1 — Supplementary Information. [file 41598_2021_95682_MOESM1_ESM.zip › SEM_photos complete/PIC0698 (1).jpg]

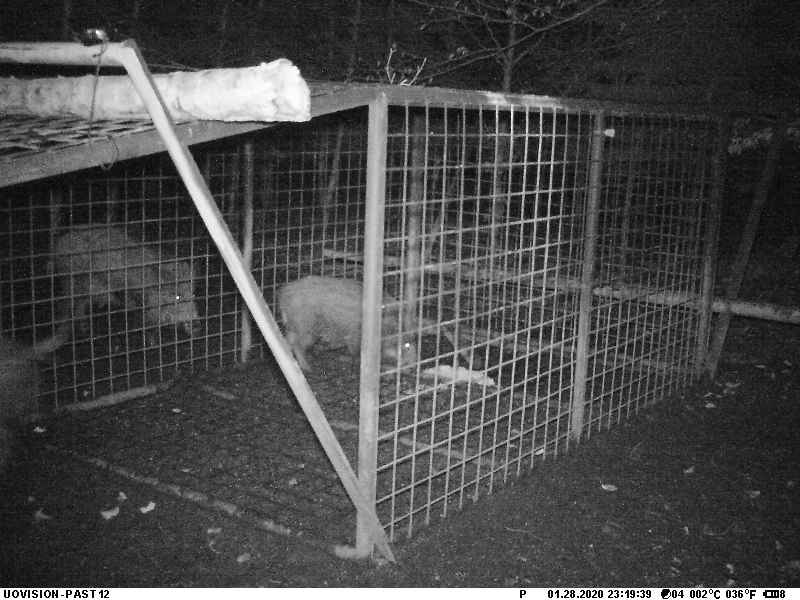

Supplement: Supplementary file 1 — Supplementary Information. [file 41598_2021_95682_MOESM1_ESM.zip › SEM_photos complete/PIC0699 (1).jpg]

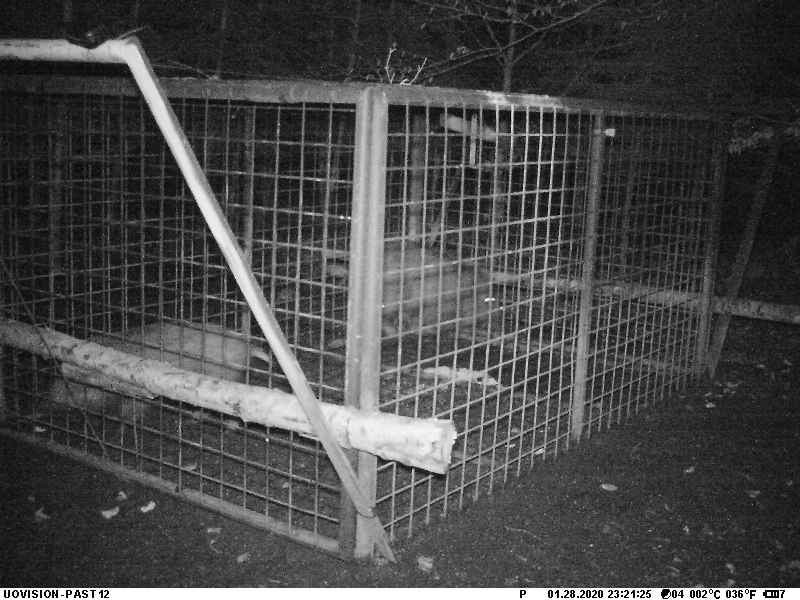

Supplement: Supplementary file 1 — Supplementary Information. [file 41598_2021_95682_MOESM1_ESM.zip › SEM_photos complete/PIC0700 (1).jpg]

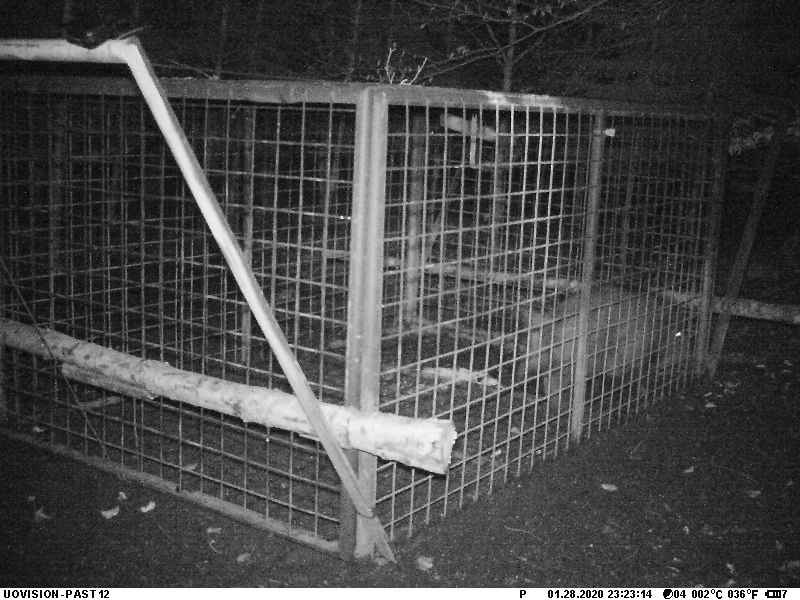

Supplement: Supplementary file 1 — Supplementary Information. [file 41598_2021_95682_MOESM1_ESM.zip › SEM_photos complete/PIC0701 (1).jpg]

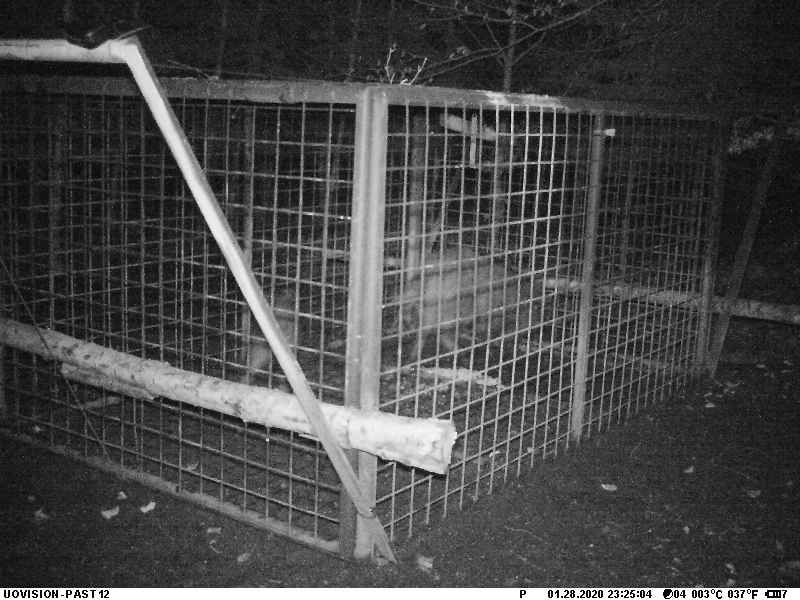

Supplement: Supplementary file 1 — Supplementary Information. [file 41598_2021_95682_MOESM1_ESM.zip › SEM_photos complete/PIC0702 (1).jpg]

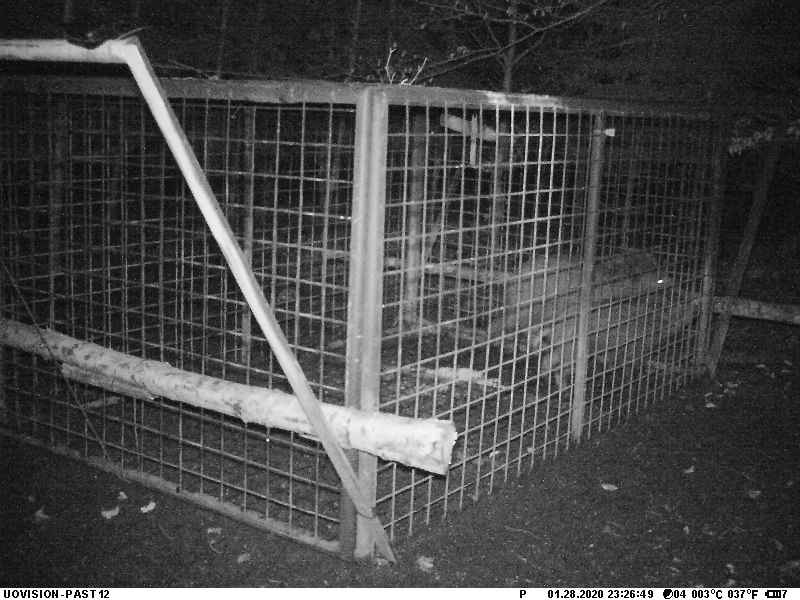

Supplement: Supplementary file 1 — Supplementary Information. [file 41598_2021_95682_MOESM1_ESM.zip › SEM_photos complete/PIC0703 (1).jpg]

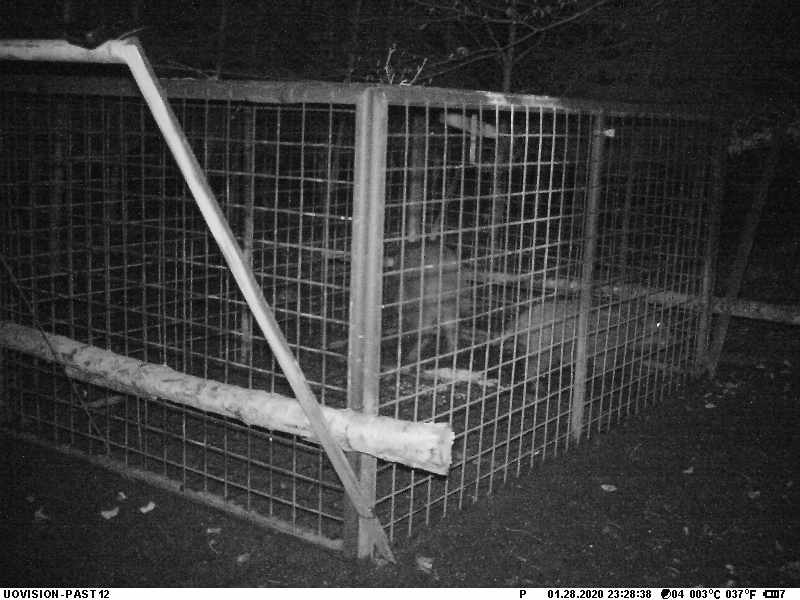

Supplement: Supplementary file 1 — Supplementary Information. [file 41598_2021_95682_MOESM1_ESM.zip › SEM_photos complete/PIC0704 (1).jpg]

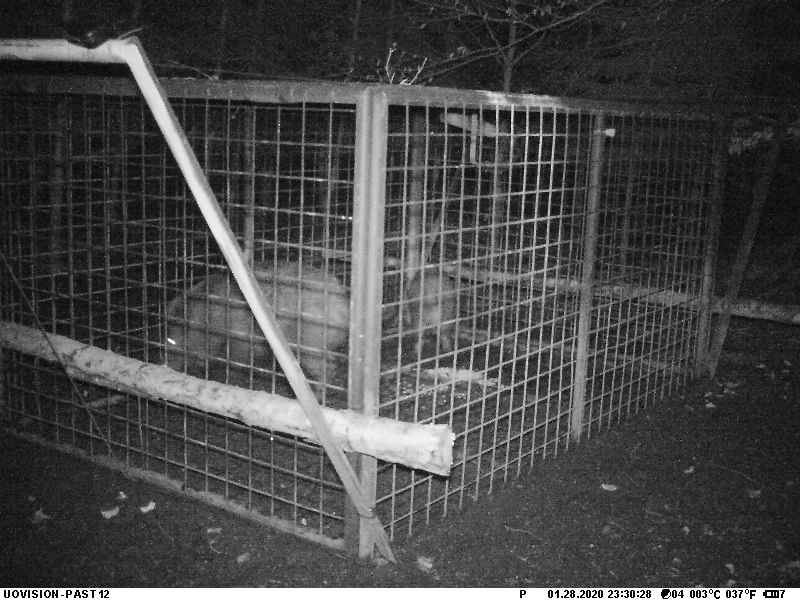

Supplement: Supplementary file 1 — Supplementary Information. [file 41598_2021_95682_MOESM1_ESM.zip › SEM_photos complete/PIC0705 (1).jpg]

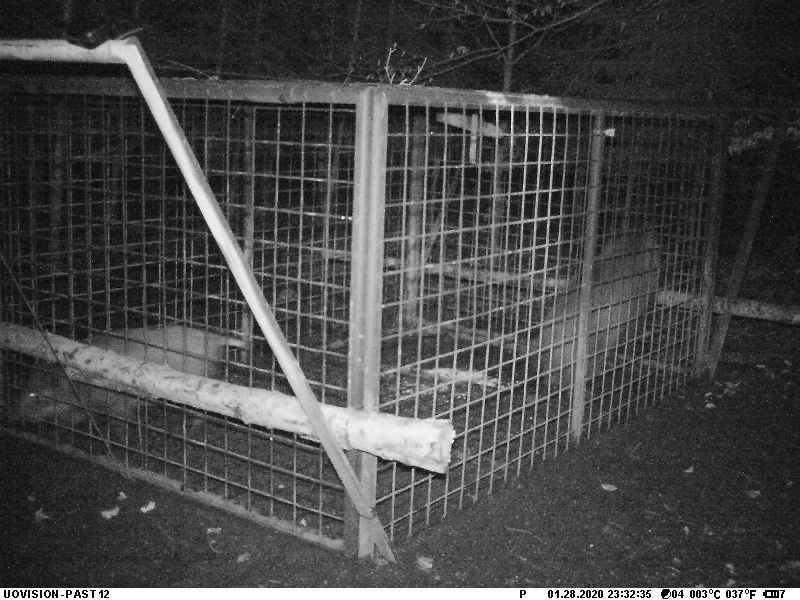

Supplement: Supplementary file 1 — Supplementary Information. [file 41598_2021_95682_MOESM1_ESM.zip › SEM_photos complete/PIC0706 (1).jpg]

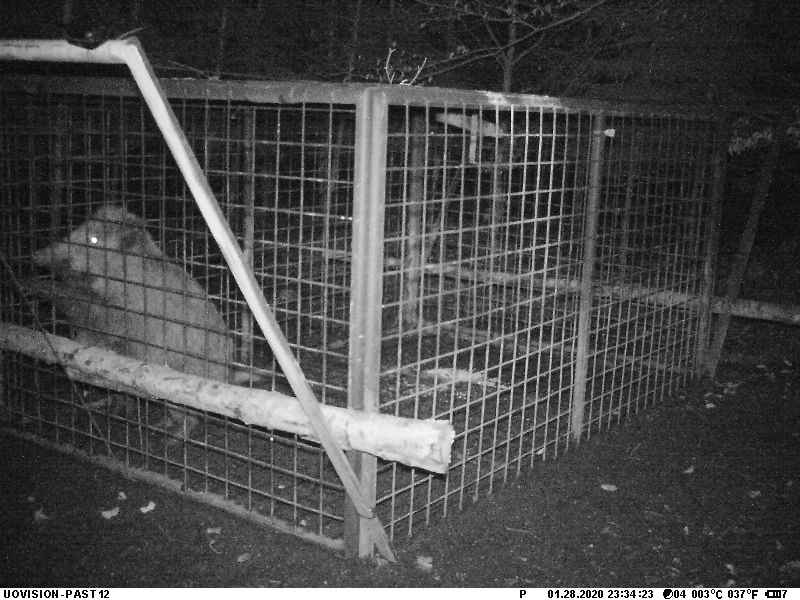

Supplement: Supplementary file 1 — Supplementary Information. [file 41598_2021_95682_MOESM1_ESM.zip › SEM_photos complete/PIC0707.jpg]

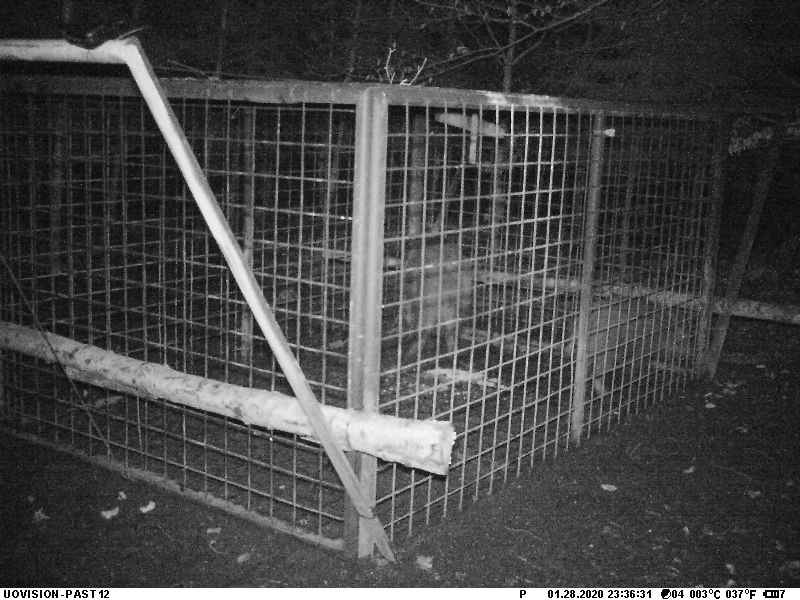

Supplement: Supplementary file 1 — Supplementary Information. [file 41598_2021_95682_MOESM1_ESM.zip › SEM_photos complete/PIC0708 (1).jpg]

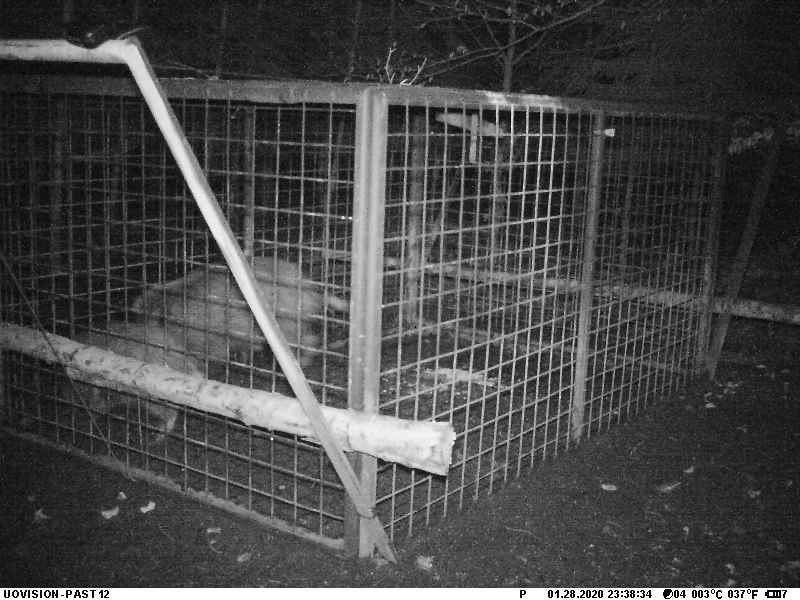

Supplement: Supplementary file 1 — Supplementary Information. [file 41598_2021_95682_MOESM1_ESM.zip › SEM_photos complete/PIC0709 (1).jpg]

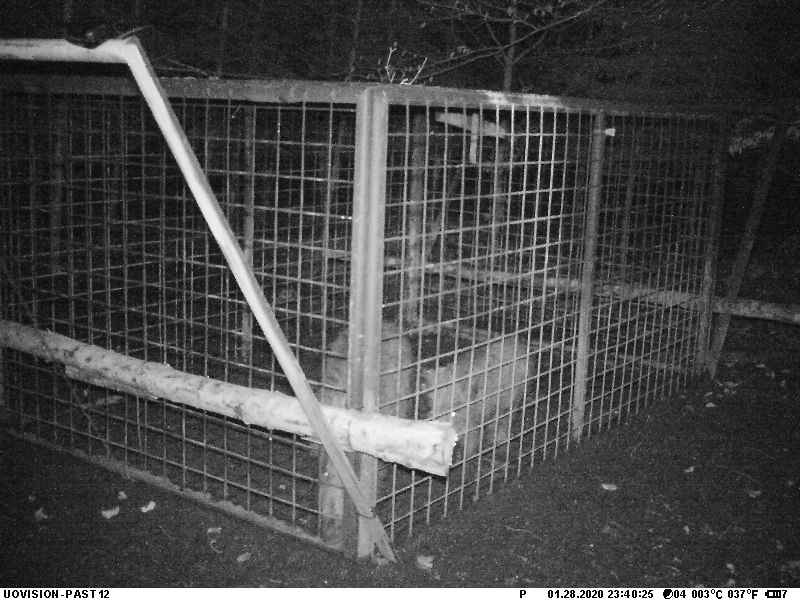

Supplement: Supplementary file 1 — Supplementary Information. [file 41598_2021_95682_MOESM1_ESM.zip › SEM_photos complete/PIC0710 (1).jpg]

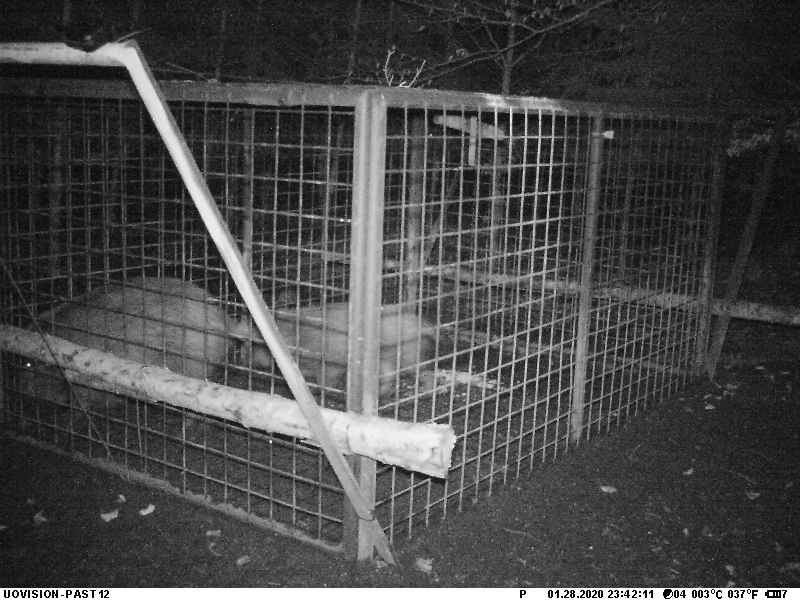

Supplement: Supplementary file 1 — Supplementary Information. [file 41598_2021_95682_MOESM1_ESM.zip › SEM_photos complete/PIC0711 (1).jpg]

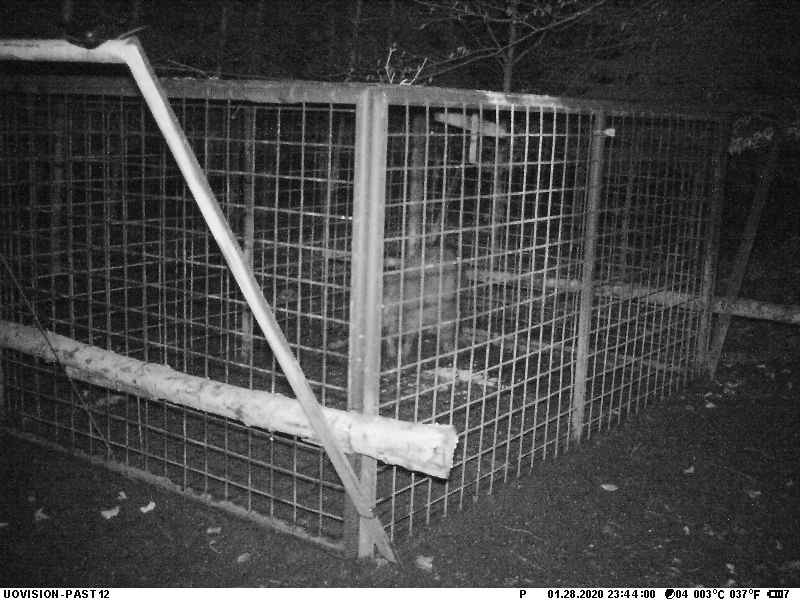

Supplement: Supplementary file 1 — Supplementary Information. [file 41598_2021_95682_MOESM1_ESM.zip › SEM_photos complete/PIC0712 (1).jpg]

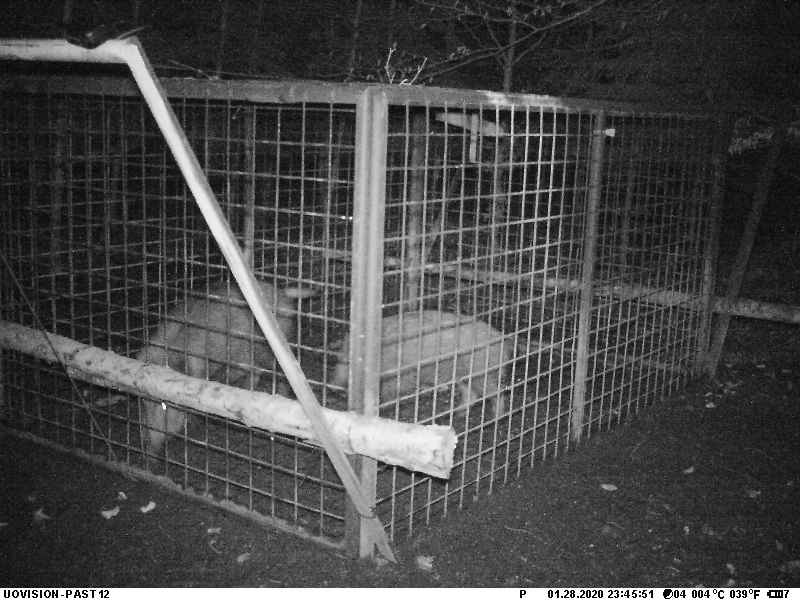

Supplement: Supplementary file 1 — Supplementary Information. [file 41598_2021_95682_MOESM1_ESM.zip › SEM_photos complete/PIC0713.jpg]

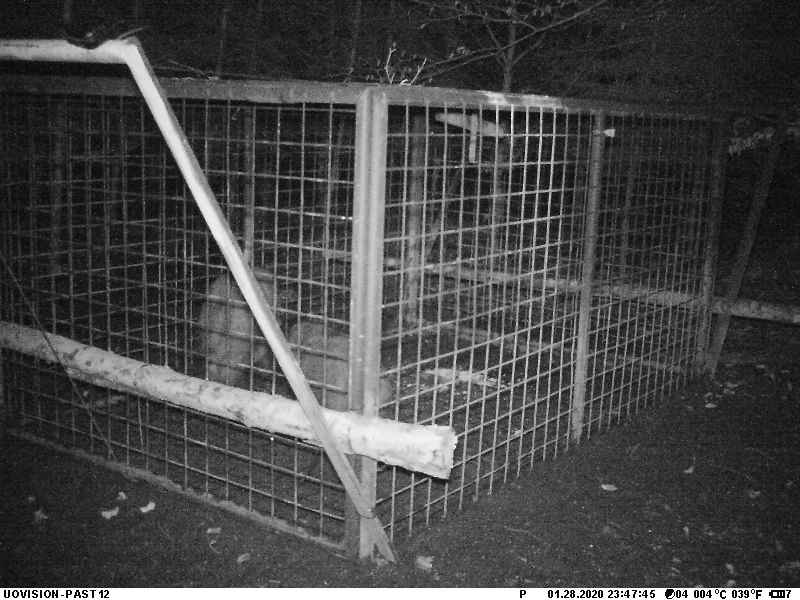

Supplement: Supplementary file 1 — Supplementary Information. [file 41598_2021_95682_MOESM1_ESM.zip › SEM_photos complete/PIC0714 (1).jpg]

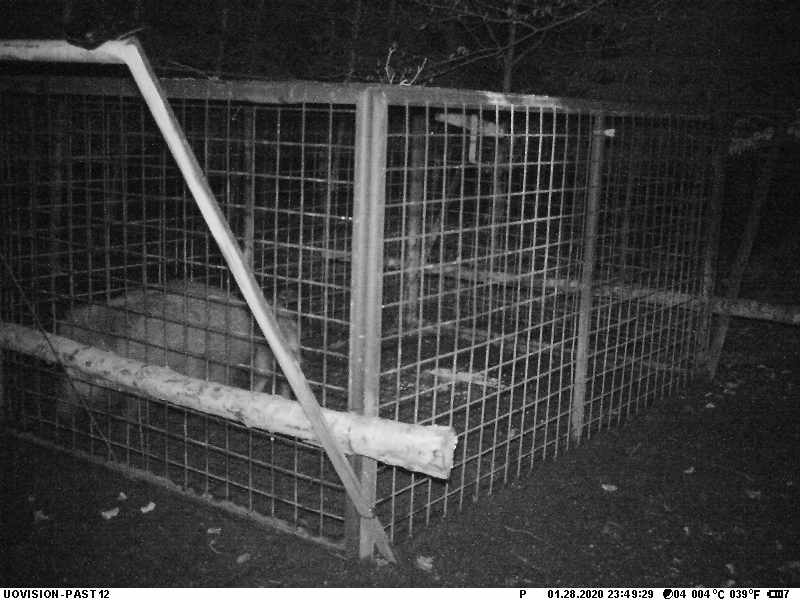

Supplement: Supplementary file 1 — Supplementary Information. [file 41598_2021_95682_MOESM1_ESM.zip › SEM_photos complete/PIC0715 (1).jpg]

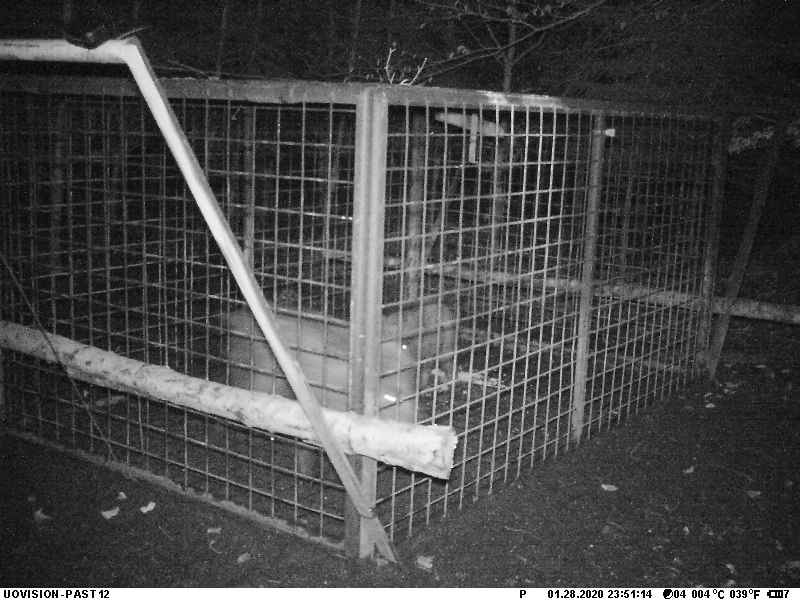

Supplement: Supplementary file 1 — Supplementary Information. [file 41598_2021_95682_MOESM1_ESM.zip › SEM_photos complete/PIC0716 (1).jpg]

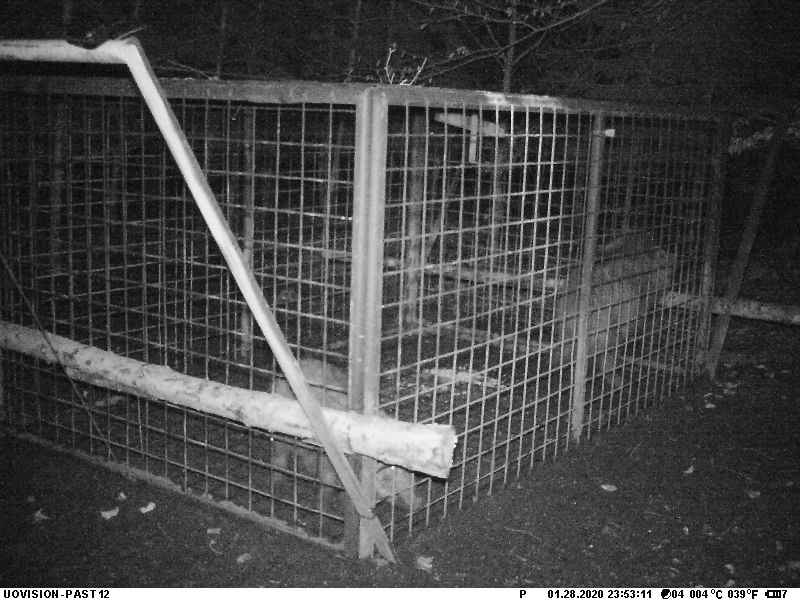

Supplement: Supplementary file 1 — Supplementary Information. [file 41598_2021_95682_MOESM1_ESM.zip › SEM_photos complete/PIC0717 (1).jpg]

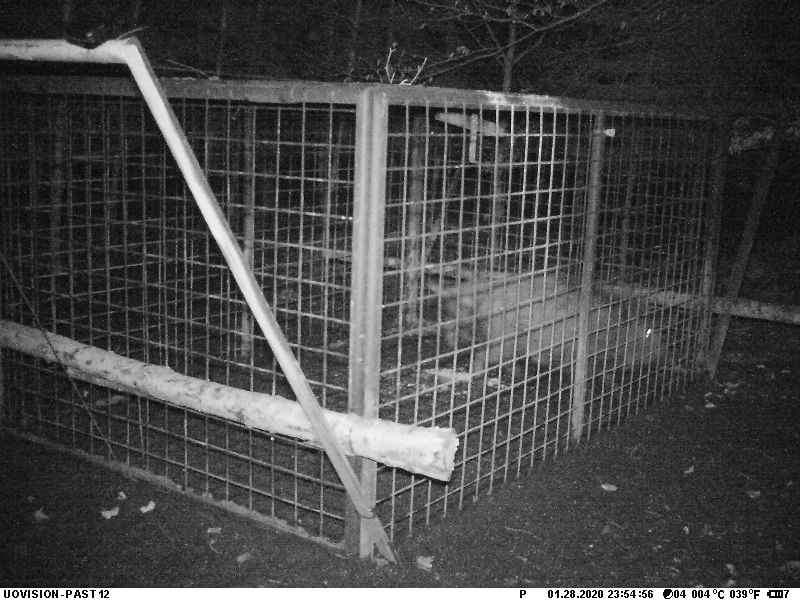

Supplement: Supplementary file 1 — Supplementary Information. [file 41598_2021_95682_MOESM1_ESM.zip › SEM_photos complete/PIC0718 (1).jpg]

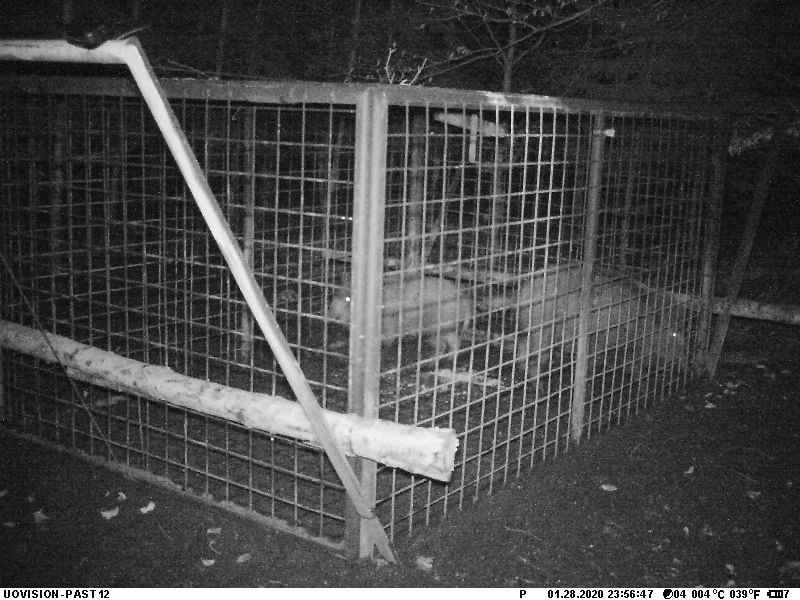

Supplement: Supplementary file 1 — Supplementary Information. [file 41598_2021_95682_MOESM1_ESM.zip › SEM_photos complete/PIC0719 (1).jpg]

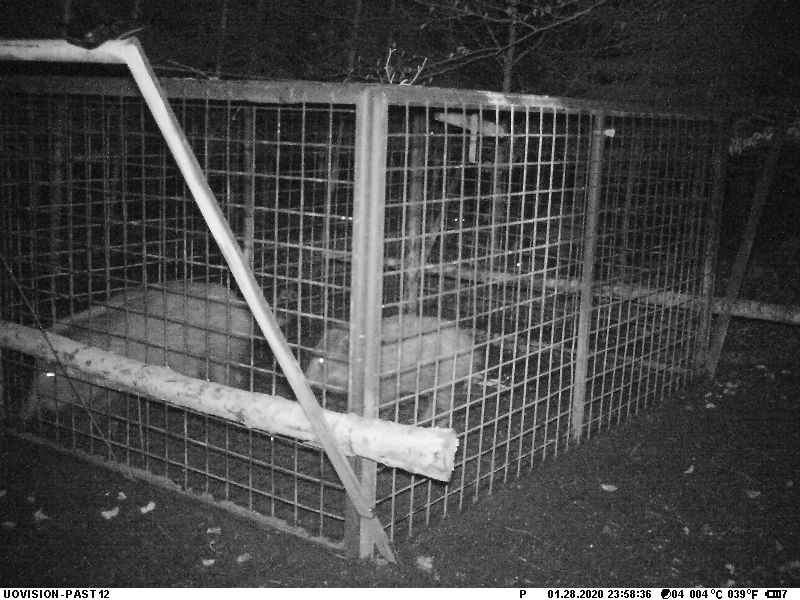

Supplement: Supplementary file 1 — Supplementary Information. [file 41598_2021_95682_MOESM1_ESM.zip › SEM_photos complete/PIC0720 (2).jpg]

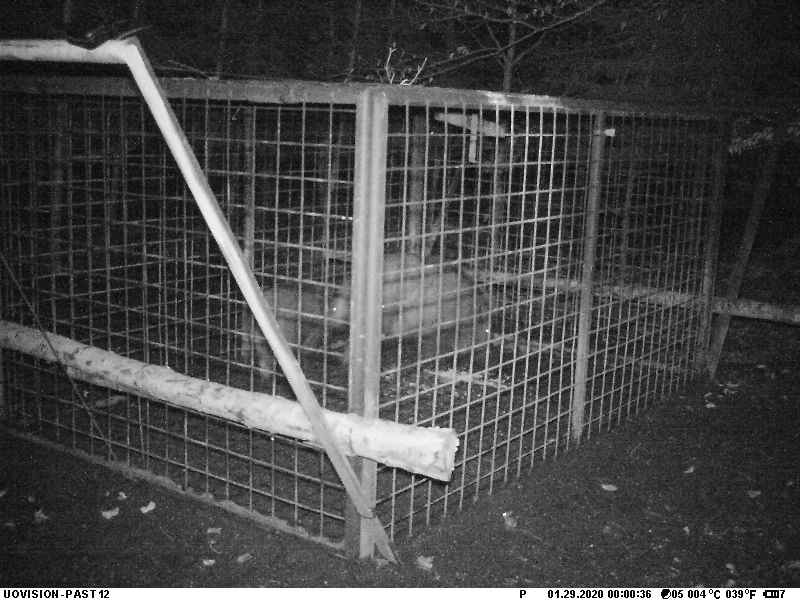

Supplement: Supplementary file 1 — Supplementary Information. [file 41598_2021_95682_MOESM1_ESM.zip › SEM_photos complete/PIC0721 (1).jpg]

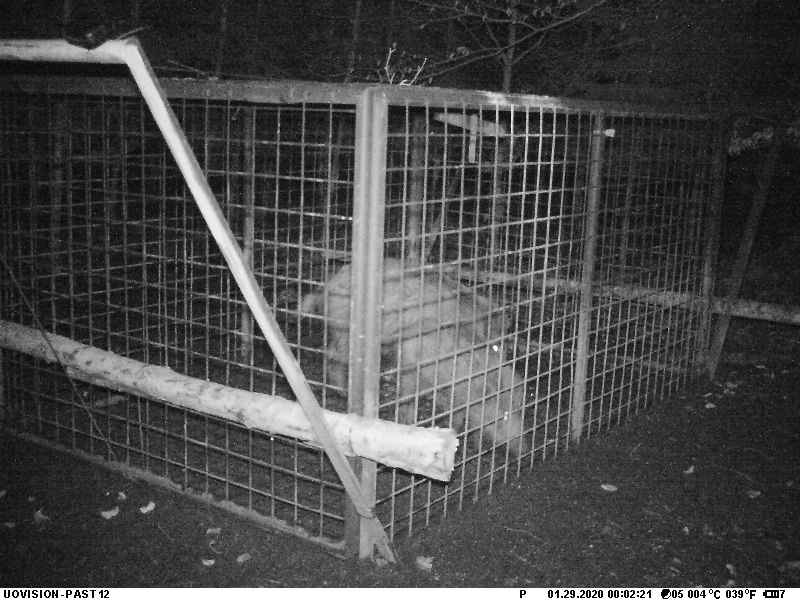

Supplement: Supplementary file 1 — Supplementary Information. [file 41598_2021_95682_MOESM1_ESM.zip › SEM_photos complete/PIC0722 (1).jpg]

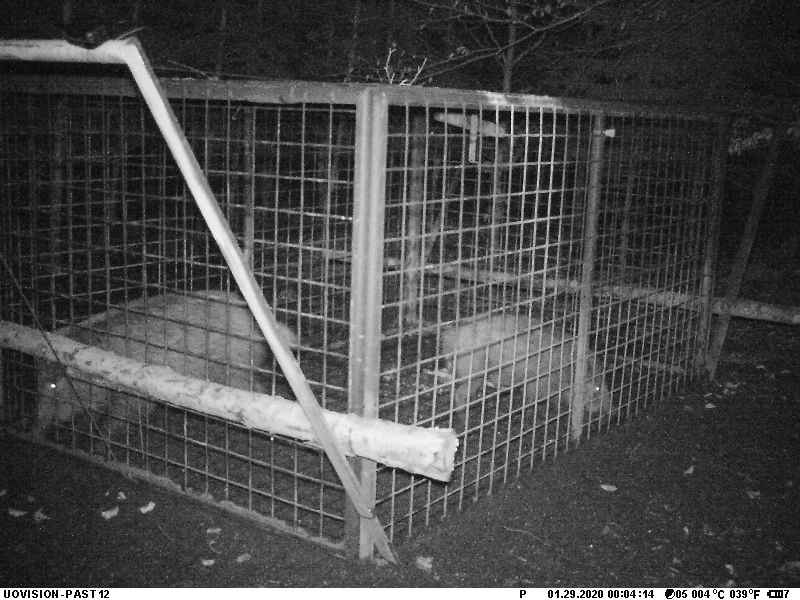

Supplement: Supplementary file 1 — Supplementary Information. [file 41598_2021_95682_MOESM1_ESM.zip › SEM_photos complete/PIC0723 (1).jpg]

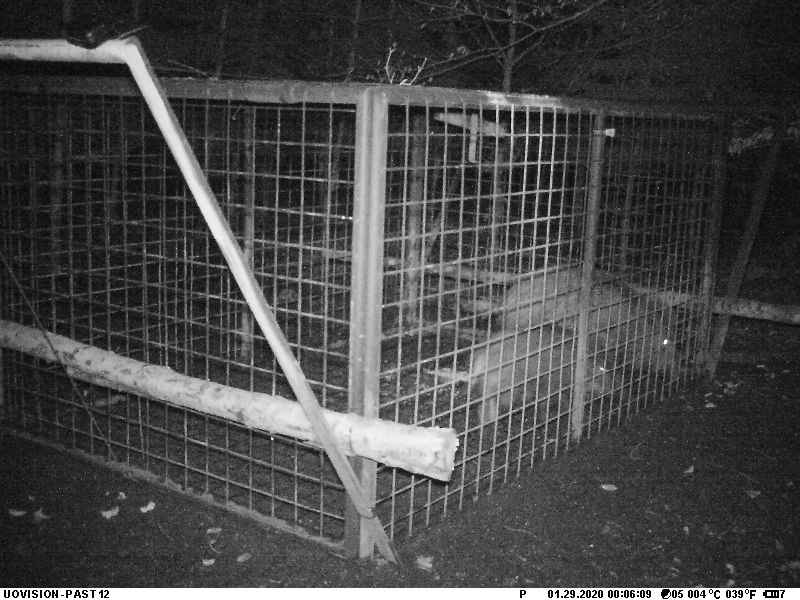

Supplement: Supplementary file 1 — Supplementary Information. [file 41598_2021_95682_MOESM1_ESM.zip › SEM_photos complete/PIC0724 (1).jpg]

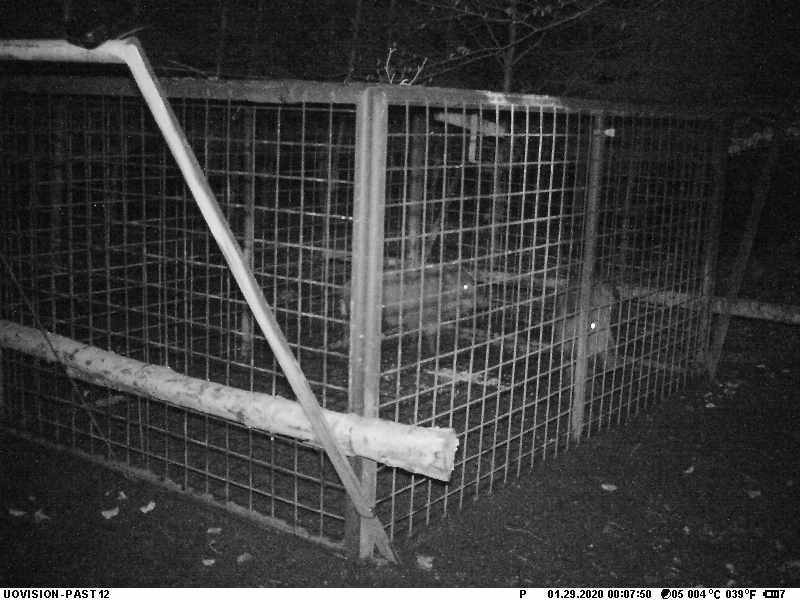

Supplement: Supplementary file 1 — Supplementary Information. [file 41598_2021_95682_MOESM1_ESM.zip › SEM_photos complete/PIC0725 (1).jpg]

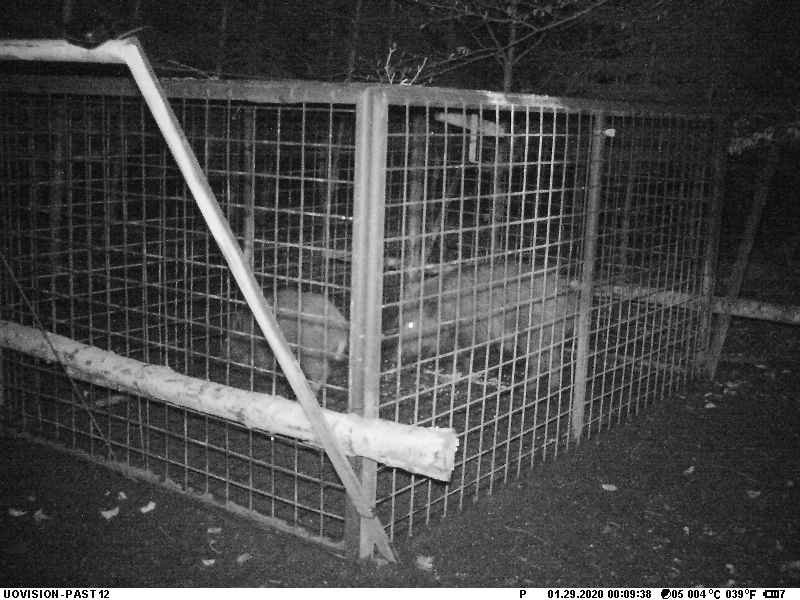

Supplement: Supplementary file 1 — Supplementary Information. [file 41598_2021_95682_MOESM1_ESM.zip › SEM_photos complete/PIC0726 (1).jpg]

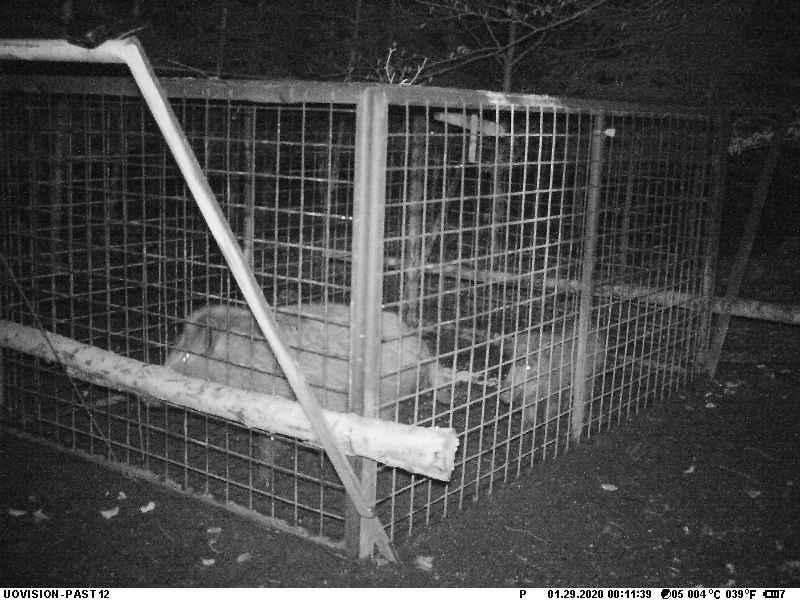

Supplement: Supplementary file 1 — Supplementary Information. [file 41598_2021_95682_MOESM1_ESM.zip › SEM_photos complete/PIC0727 (1).jpg]

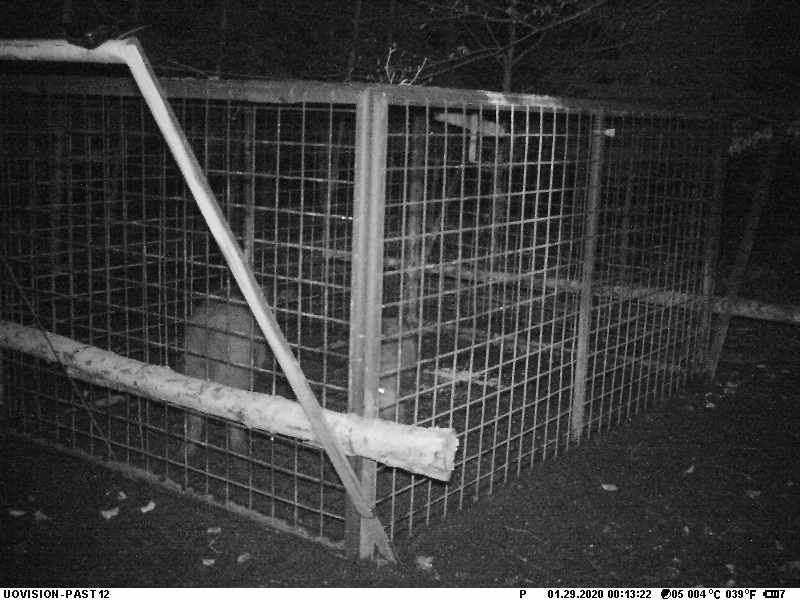

Supplement: Supplementary file 1 — Supplementary Information. [file 41598_2021_95682_MOESM1_ESM.zip › SEM_photos complete/PIC0728.jpg]

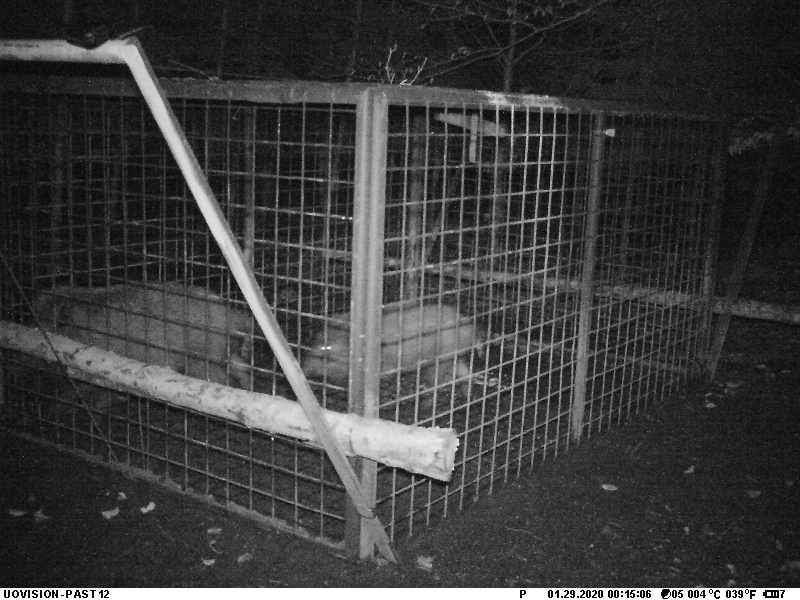

Supplement: Supplementary file 1 — Supplementary Information. [file 41598_2021_95682_MOESM1_ESM.zip › SEM_photos complete/PIC0729 (1).jpg]

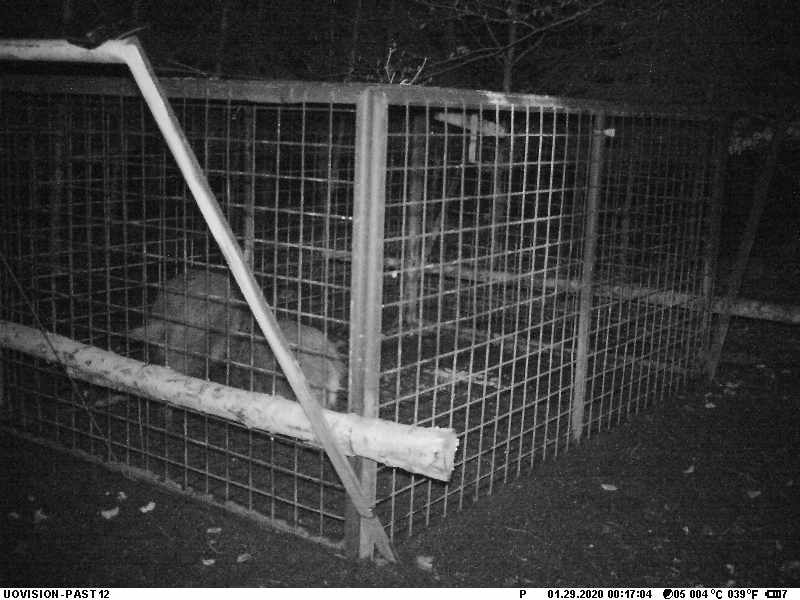

Supplement: Supplementary file 1 — Supplementary Information. [file 41598_2021_95682_MOESM1_ESM.zip › SEM_photos complete/PIC0730 (1).jpg]

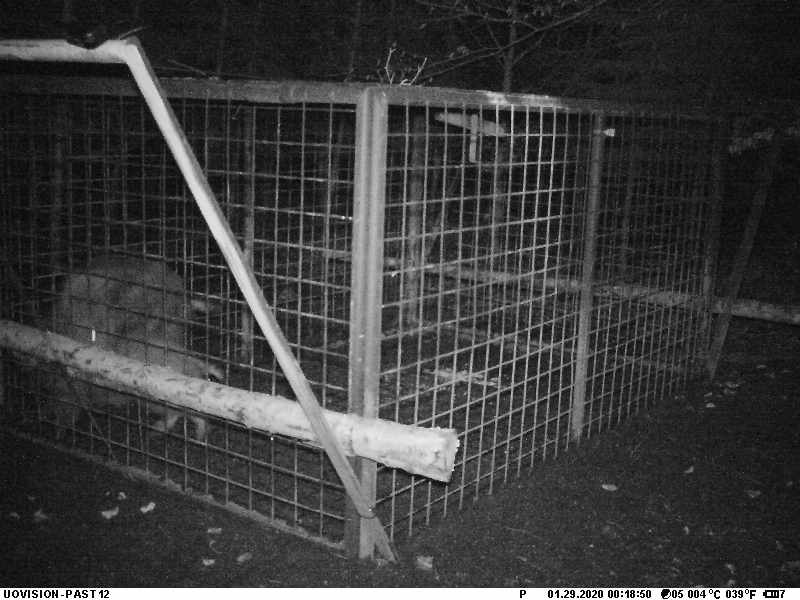

Supplement: Supplementary file 1 — Supplementary Information. [file 41598_2021_95682_MOESM1_ESM.zip › SEM_photos complete/PIC0731.jpg]

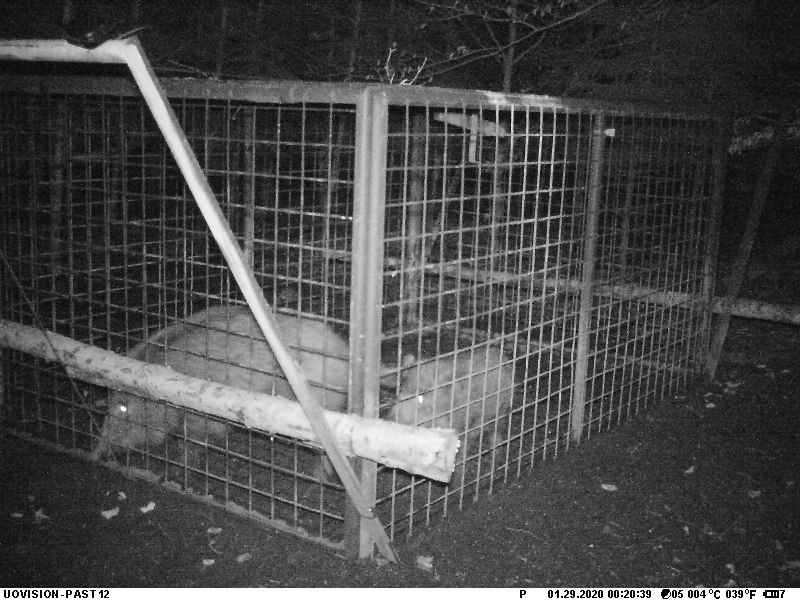

Supplement: Supplementary file 1 — Supplementary Information. [file 41598_2021_95682_MOESM1_ESM.zip › SEM_photos complete/PIC0732.jpg]

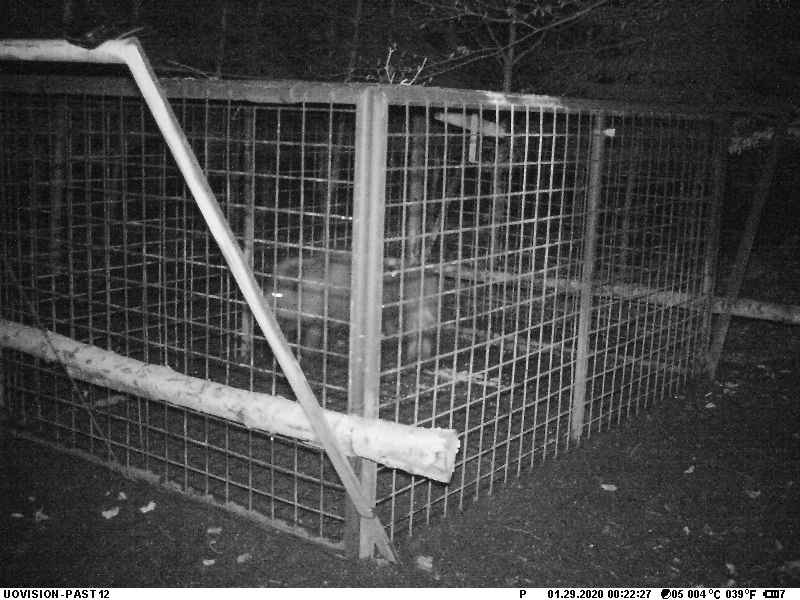

Supplement: Supplementary file 1 — Supplementary Information. [file 41598_2021_95682_MOESM1_ESM.zip › SEM_photos complete/PIC0733.jpg]

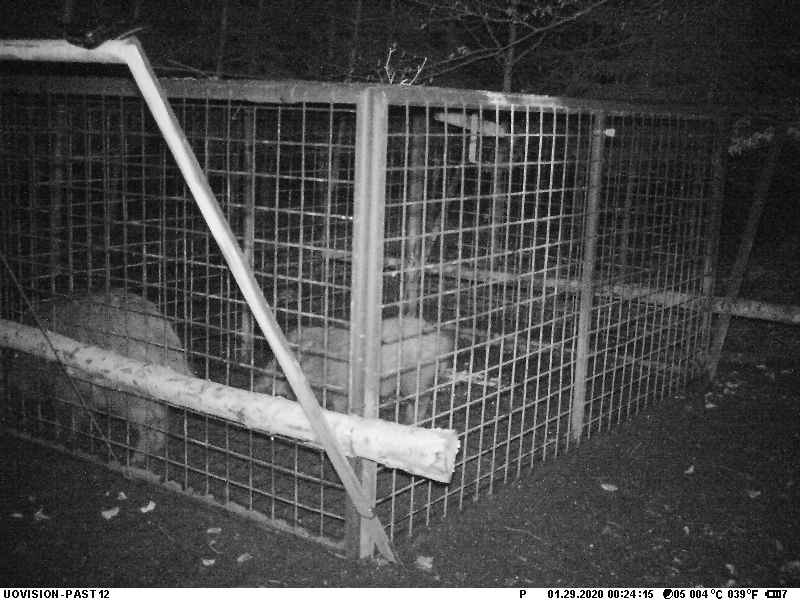

Supplement: Supplementary file 1 — Supplementary Information. [file 41598_2021_95682_MOESM1_ESM.zip › SEM_photos complete/PIC0734.jpg]

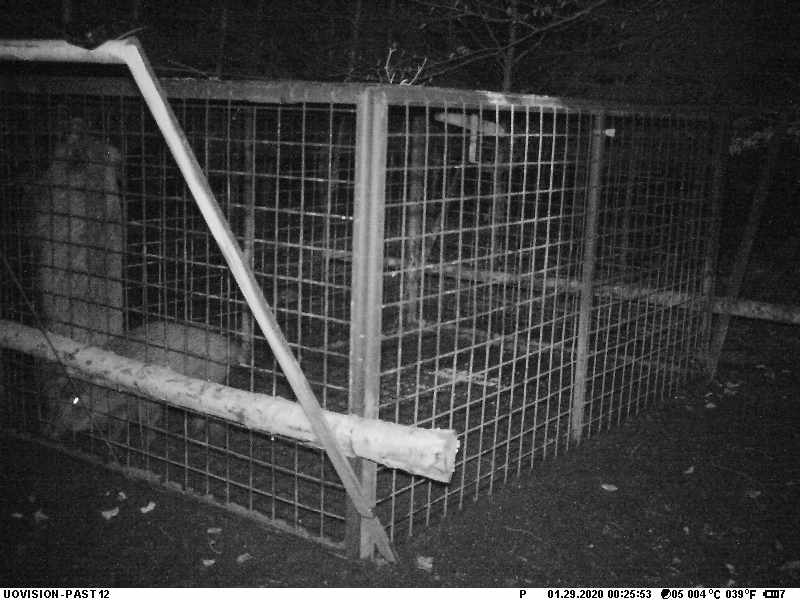

Supplement: Supplementary file 1 — Supplementary Information. [file 41598_2021_95682_MOESM1_ESM.zip › SEM_photos complete/PIC0735.jpg]

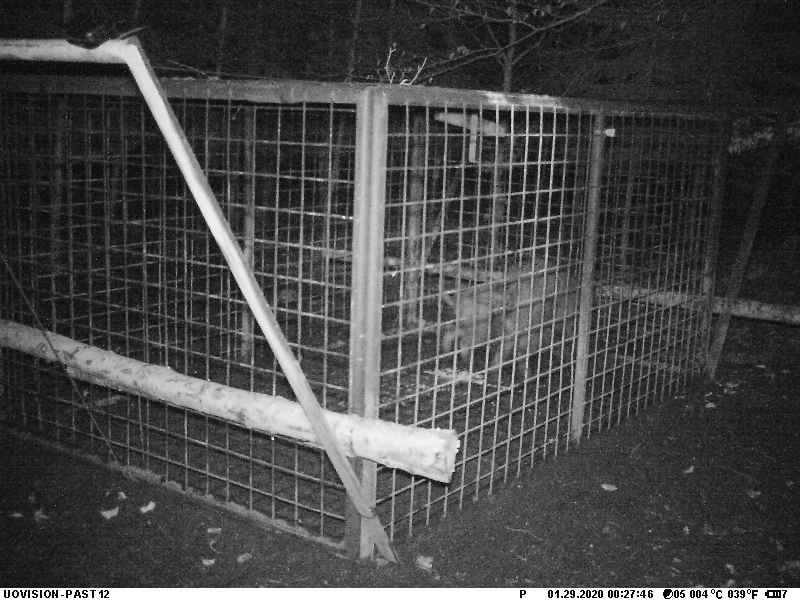

Supplement: Supplementary file 1 — Supplementary Information. [file 41598_2021_95682_MOESM1_ESM.zip › SEM_photos complete/PIC0736.jpg]

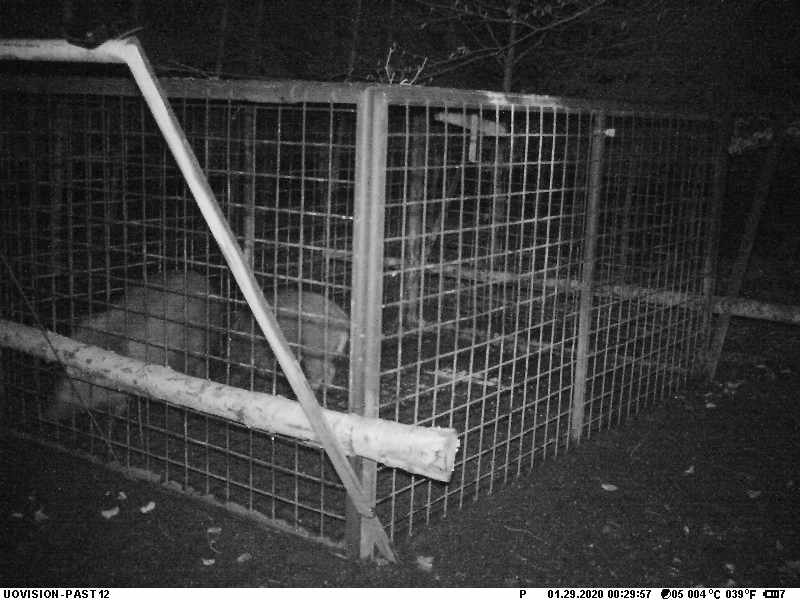

Supplement: Supplementary file 1 — Supplementary Information. [file 41598_2021_95682_MOESM1_ESM.zip › SEM_photos complete/PIC0737.jpg]

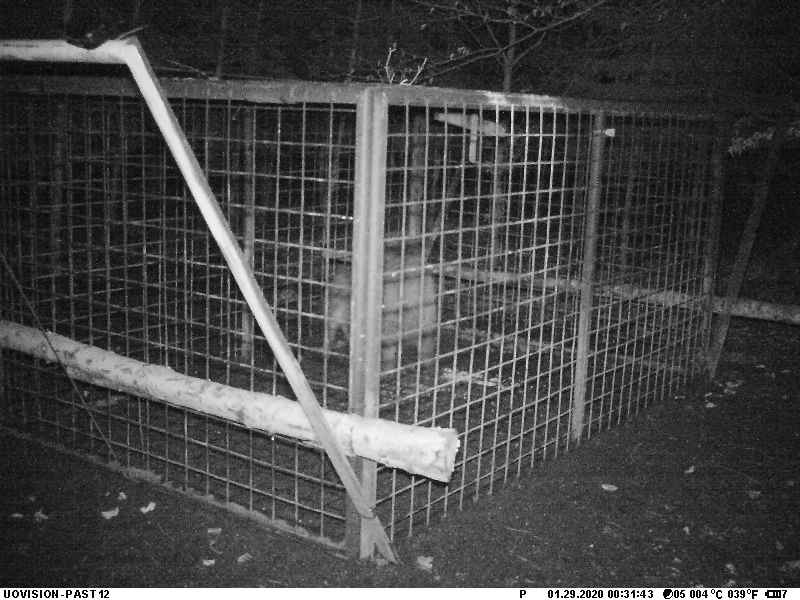

Supplement: Supplementary file 1 — Supplementary Information. [file 41598_2021_95682_MOESM1_ESM.zip › SEM_photos complete/PIC0738.jpg]

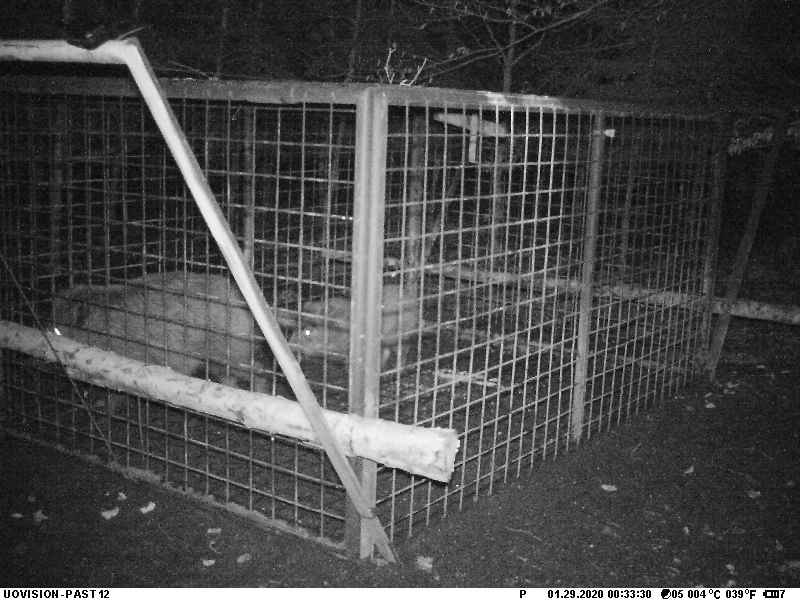

Supplement: Supplementary file 1 — Supplementary Information. [file 41598_2021_95682_MOESM1_ESM.zip › SEM_photos complete/PIC0739.jpg]

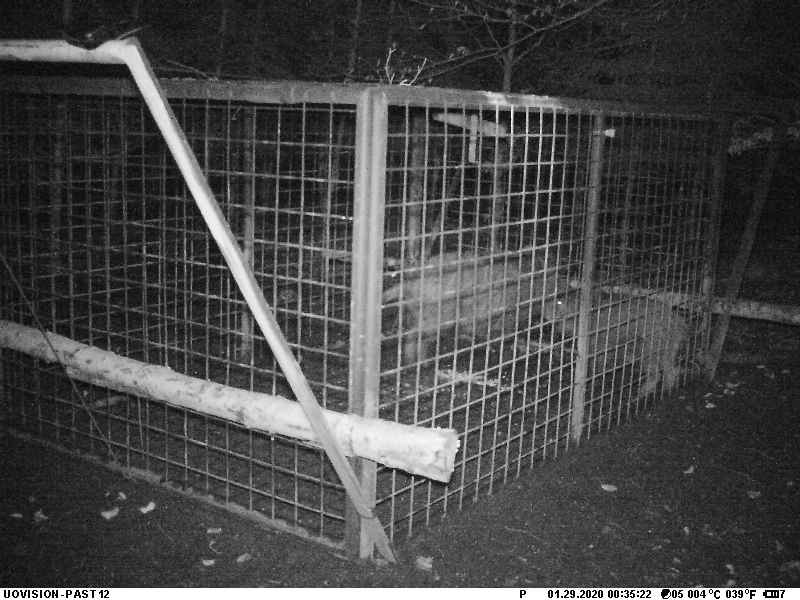

Supplement: Supplementary file 1 — Supplementary Information. [file 41598_2021_95682_MOESM1_ESM.zip › SEM_photos complete/PIC0740.jpg]

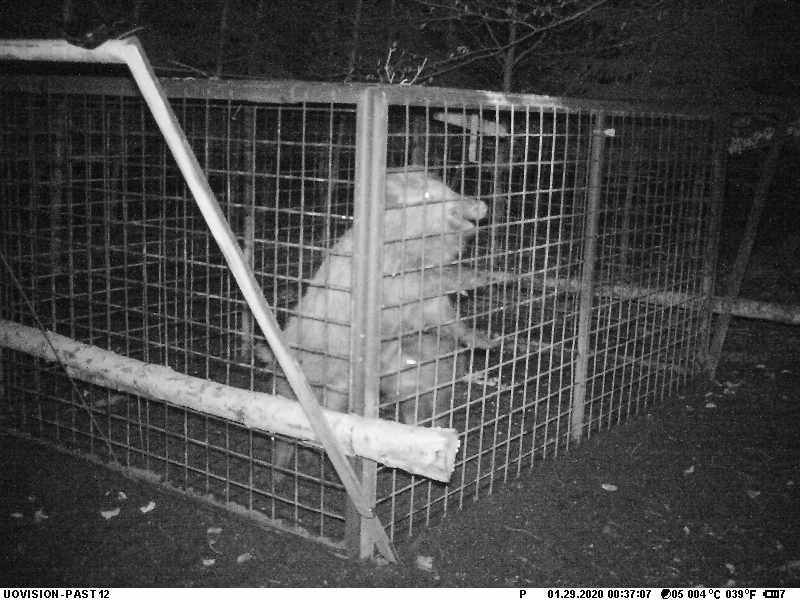

Supplement: Supplementary file 1 — Supplementary Information. [file 41598_2021_95682_MOESM1_ESM.zip › SEM_photos complete/PIC0741.jpg]

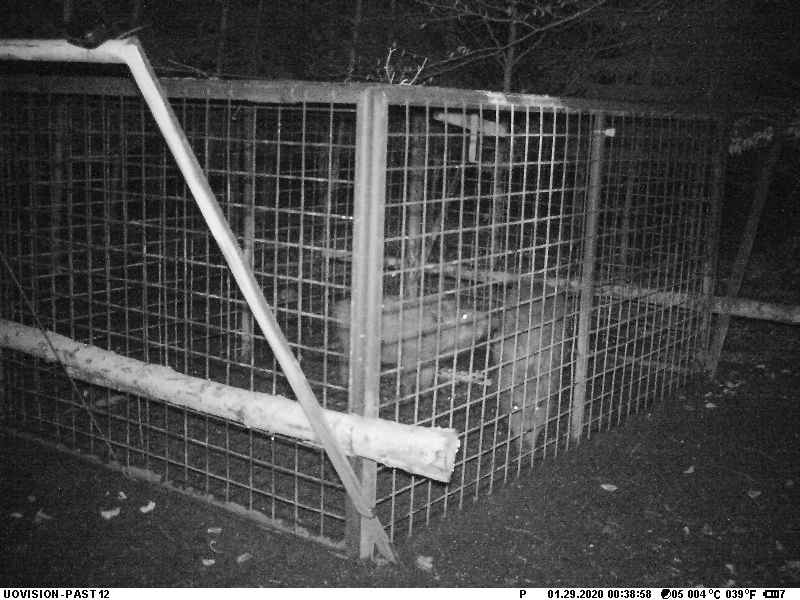

Supplement: Supplementary file 1 — Supplementary Information. [file 41598_2021_95682_MOESM1_ESM.zip › SEM_photos complete/PIC0742.jpg]

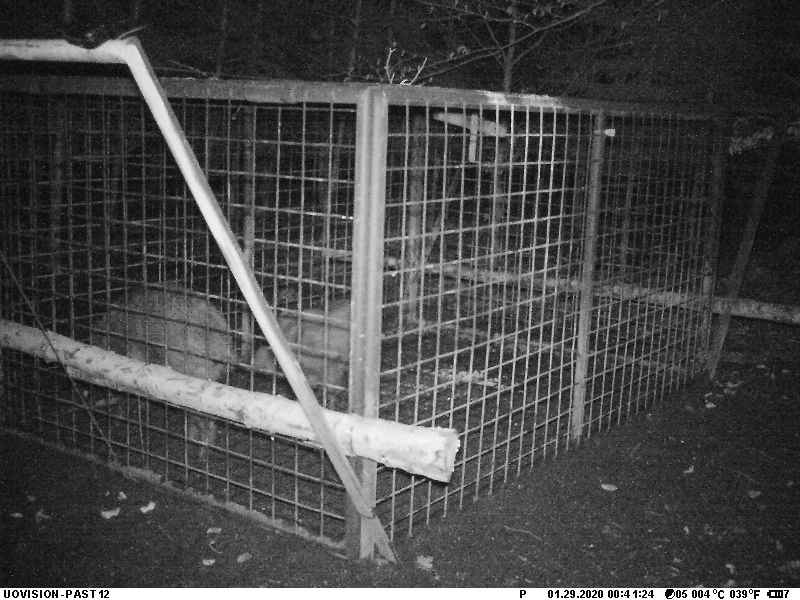

Supplement: Supplementary file 1 — Supplementary Information. [file 41598_2021_95682_MOESM1_ESM.zip › SEM_photos complete/PIC0743.jpg]

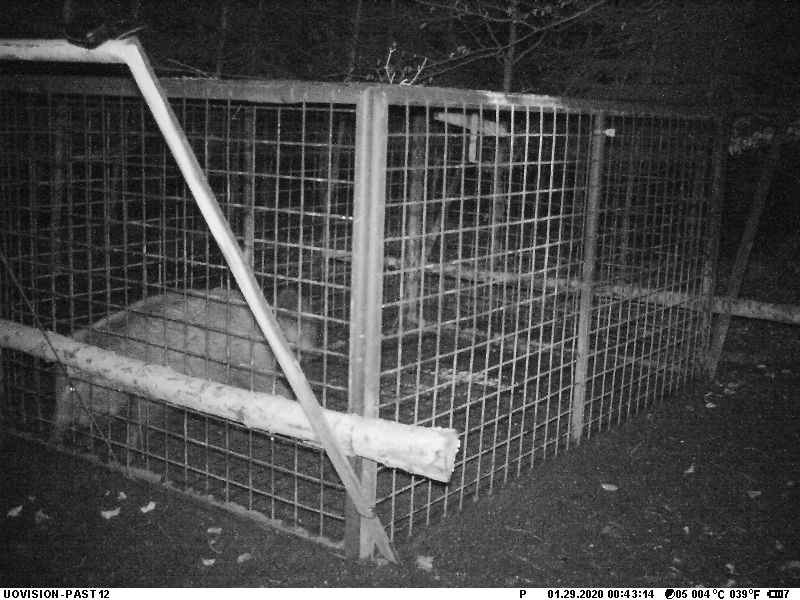

Supplement: Supplementary file 1 — Supplementary Information. [file 41598_2021_95682_MOESM1_ESM.zip › SEM_photos complete/PIC0744.jpg]

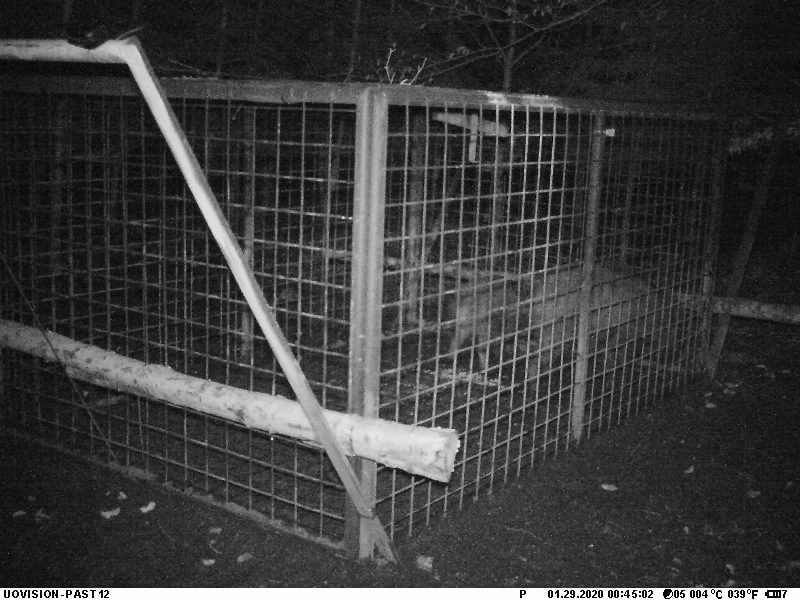

Supplement: Supplementary file 1 — Supplementary Information. [file 41598_2021_95682_MOESM1_ESM.zip › SEM_photos complete/PIC0745.jpg]

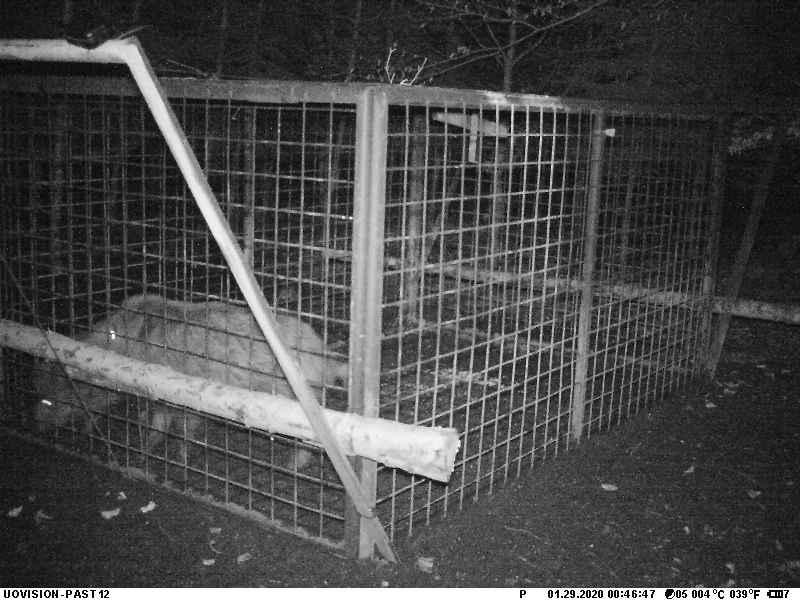

Supplement: Supplementary file 1 — Supplementary Information. [file 41598_2021_95682_MOESM1_ESM.zip › SEM_photos complete/PIC0746.jpg]

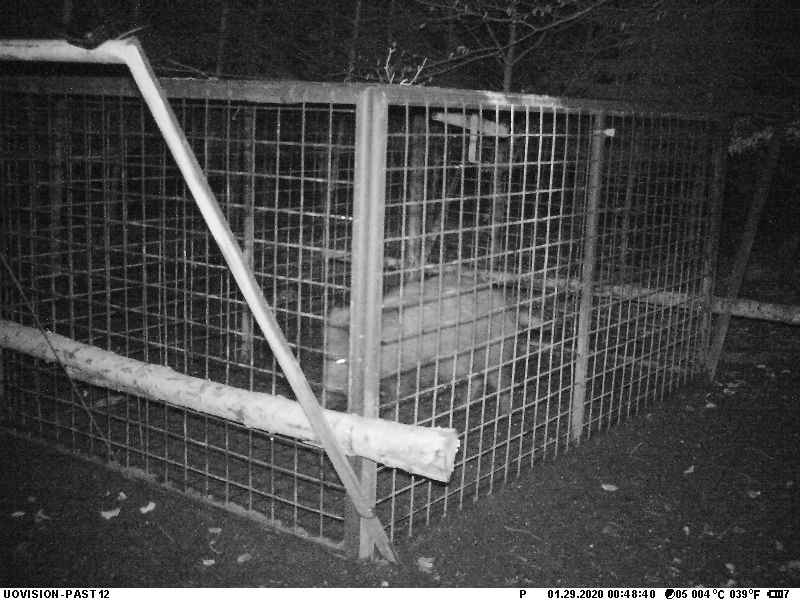

Supplement: Supplementary file 1 — Supplementary Information. [file 41598_2021_95682_MOESM1_ESM.zip › SEM_photos complete/PIC0747.jpg]

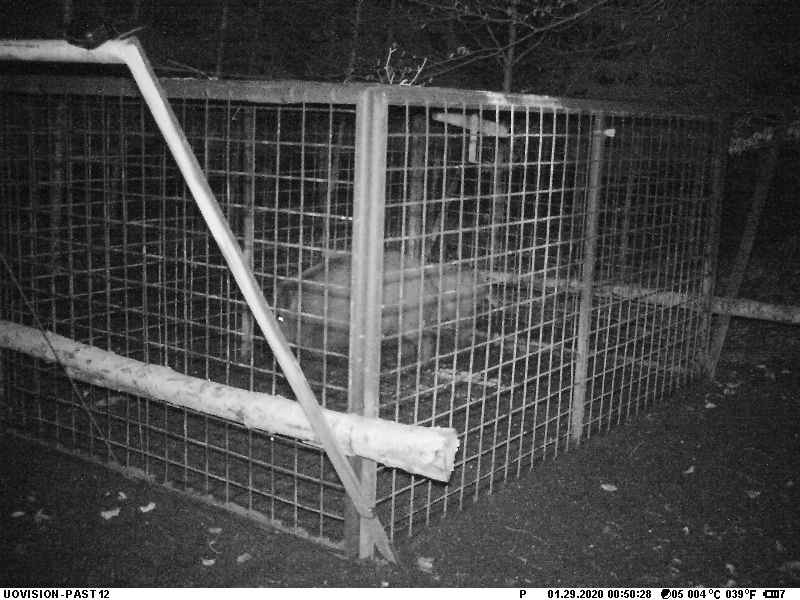

Supplement: Supplementary file 1 — Supplementary Information. [file 41598_2021_95682_MOESM1_ESM.zip › SEM_photos complete/PIC0748.jpg]

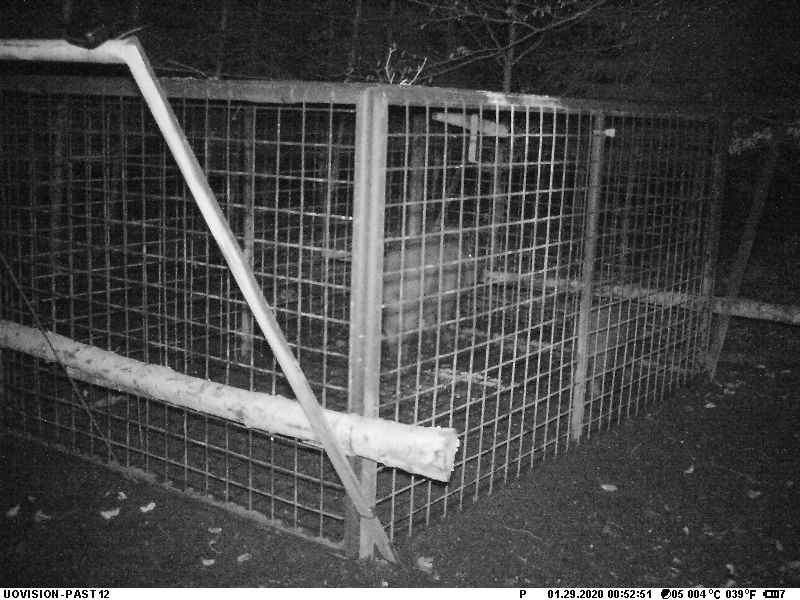

Supplement: Supplementary file 1 — Supplementary Information. [file 41598_2021_95682_MOESM1_ESM.zip › SEM_photos complete/PIC0749.jpg]

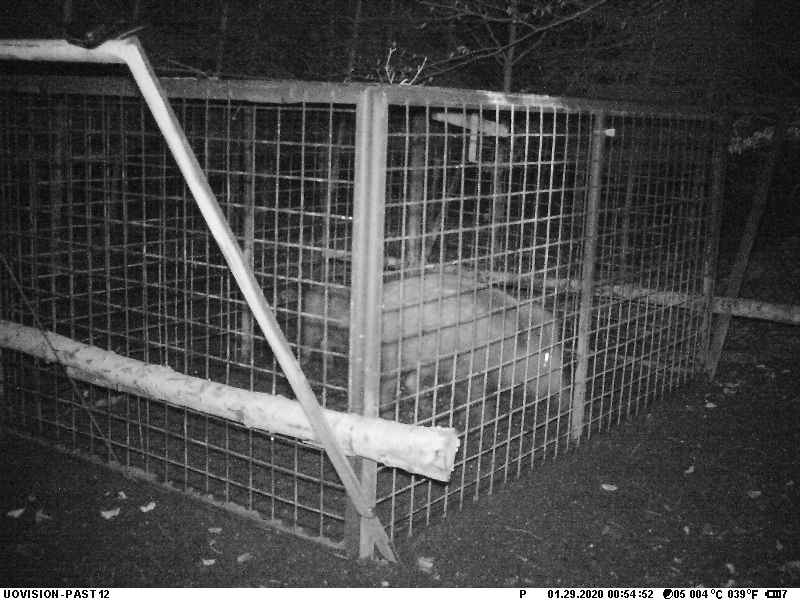

Supplement: Supplementary file 1 — Supplementary Information. [file 41598_2021_95682_MOESM1_ESM.zip › SEM_photos complete/PIC0750.jpg]

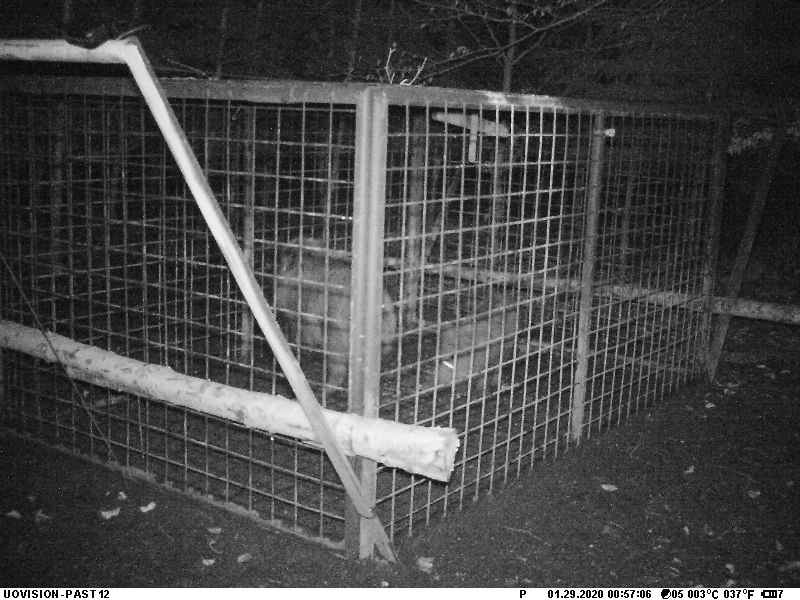

Supplement: Supplementary file 1 — Supplementary Information. [file 41598_2021_95682_MOESM1_ESM.zip › SEM_photos complete/PIC0751.jpg]

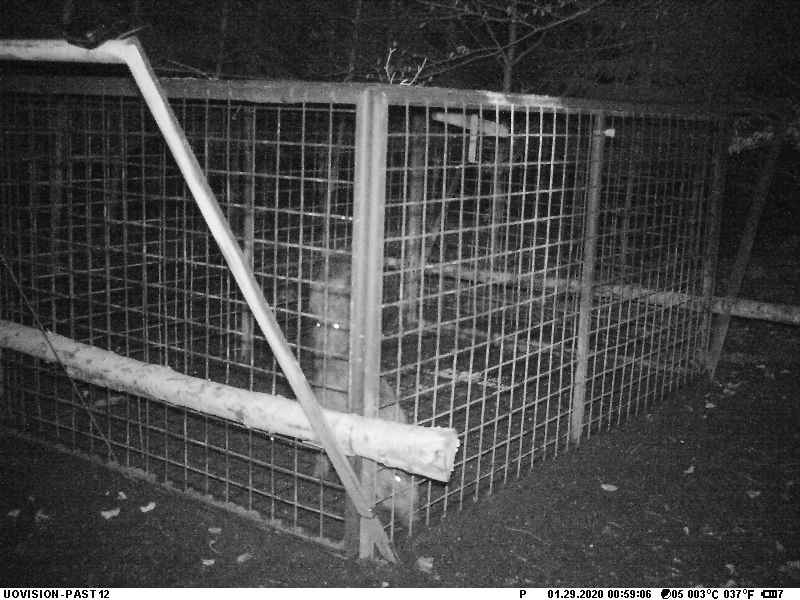

Supplement: Supplementary file 1 — Supplementary Information. [file 41598_2021_95682_MOESM1_ESM.zip › SEM_photos complete/PIC0752.jpg]

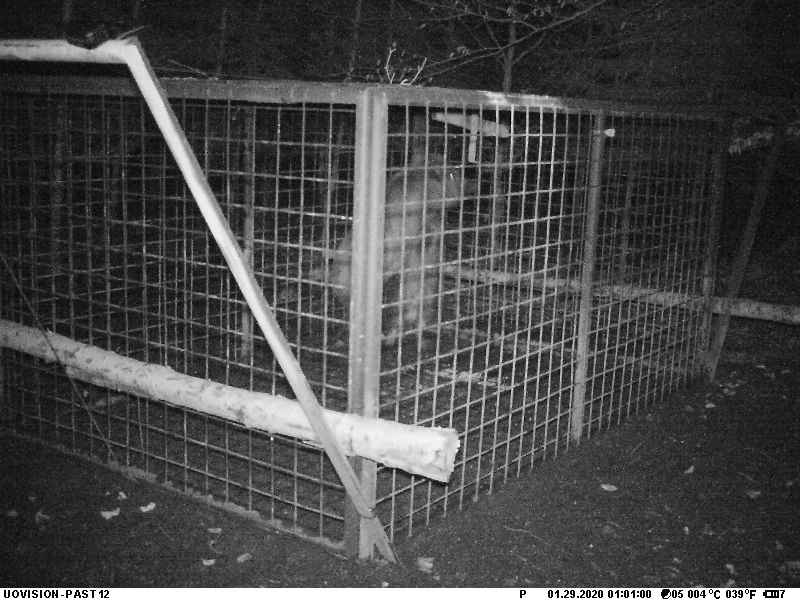

Supplement: Supplementary file 1 — Supplementary Information. [file 41598_2021_95682_MOESM1_ESM.zip › SEM_photos complete/PIC0753.jpg]

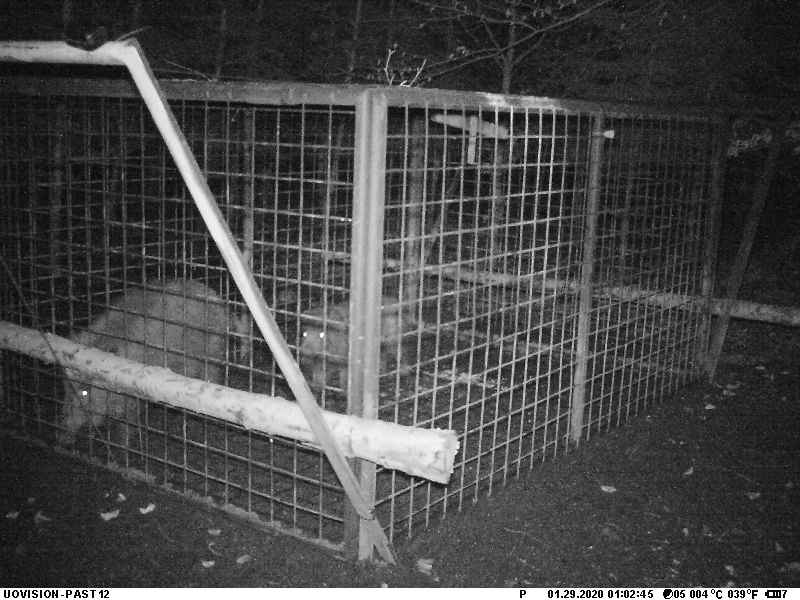

Supplement: Supplementary file 1 — Supplementary Information. [file 41598_2021_95682_MOESM1_ESM.zip › SEM_photos complete/PIC0754.jpg]

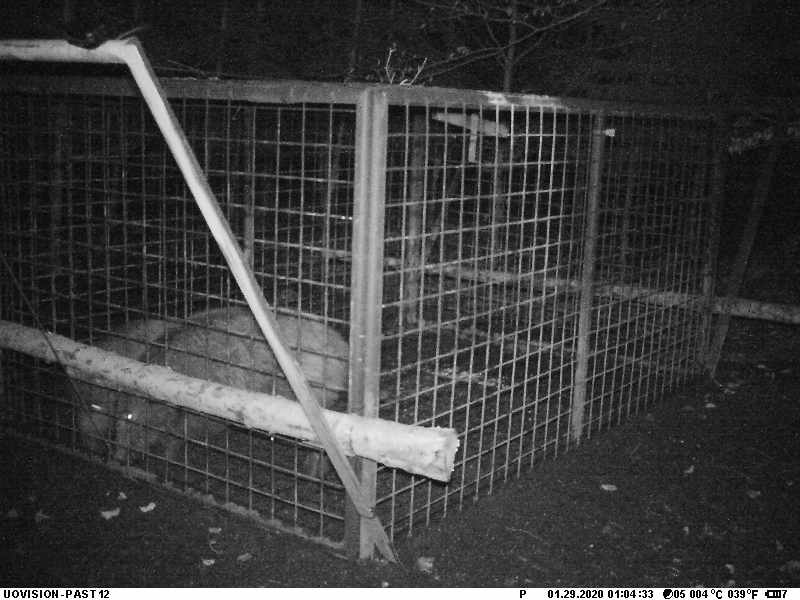

Supplement: Supplementary file 1 — Supplementary Information. [file 41598_2021_95682_MOESM1_ESM.zip › SEM_photos complete/PIC0755.jpg]

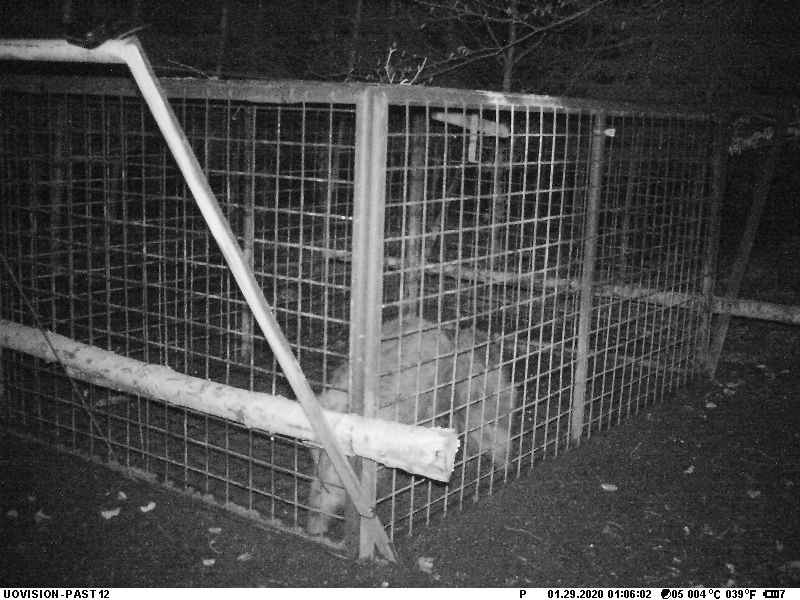

Supplement: Supplementary file 1 — Supplementary Information. [file 41598_2021_95682_MOESM1_ESM.zip › SEM_photos complete/PIC0756.jpg]

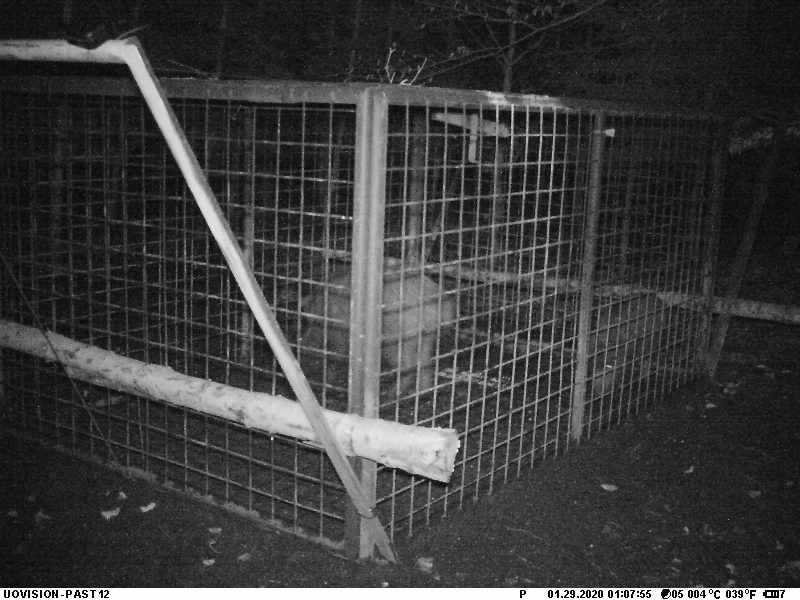

Supplement: Supplementary file 1 — Supplementary Information. [file 41598_2021_95682_MOESM1_ESM.zip › SEM_photos complete/PIC0757.jpg]

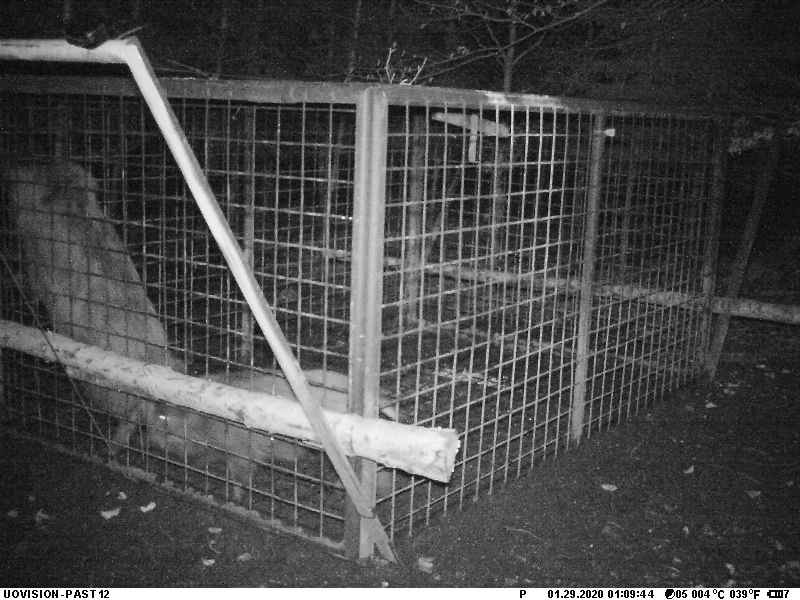

Supplement: Supplementary file 1 — Supplementary Information. [file 41598_2021_95682_MOESM1_ESM.zip › SEM_photos complete/PIC0758.jpg]

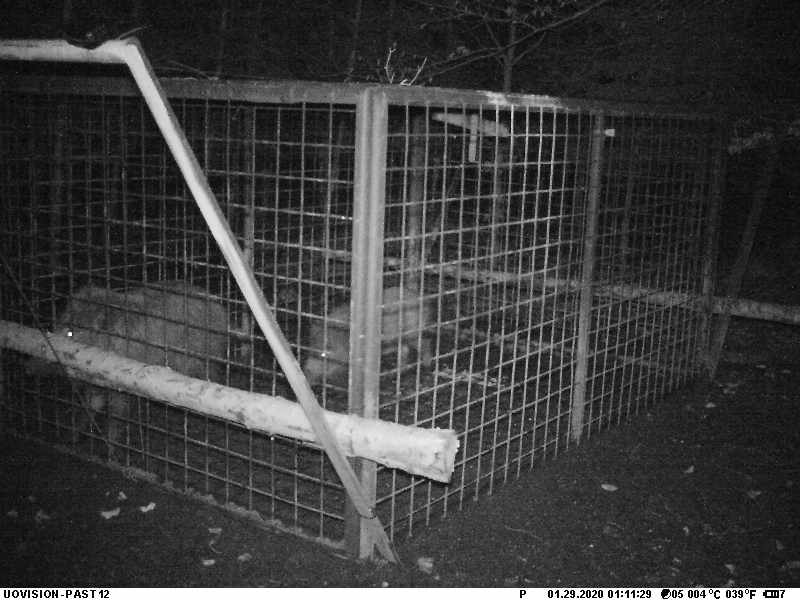

Supplement: Supplementary file 1 — Supplementary Information. [file 41598_2021_95682_MOESM1_ESM.zip › SEM_photos complete/PIC0759.jpg]

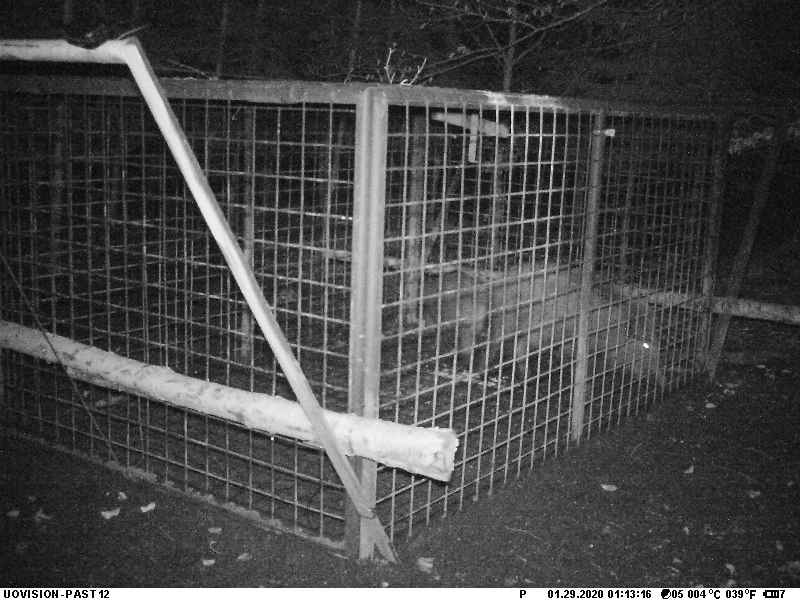

Supplement: Supplementary file 1 — Supplementary Information. [file 41598_2021_95682_MOESM1_ESM.zip › SEM_photos complete/PIC0760.jpg]

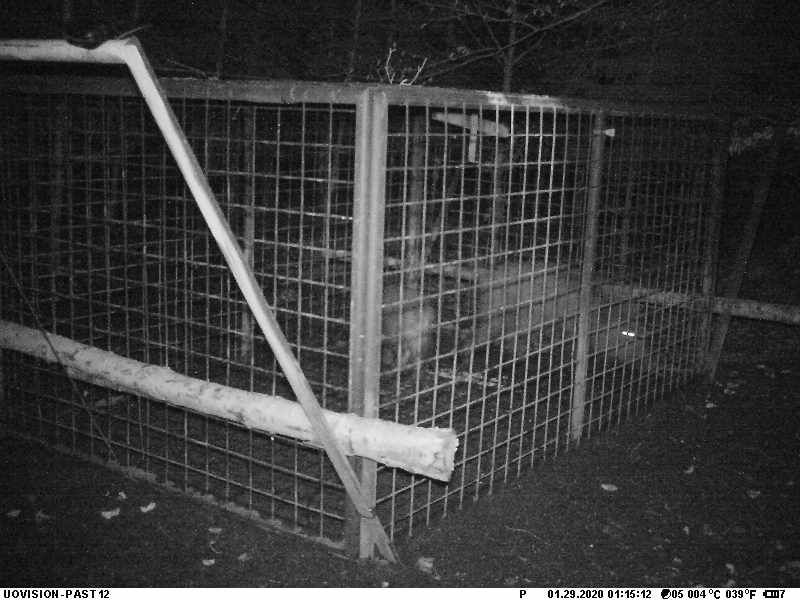

Supplement: Supplementary file 1 — Supplementary Information. [file 41598_2021_95682_MOESM1_ESM.zip › SEM_photos complete/PIC0761.jpg]

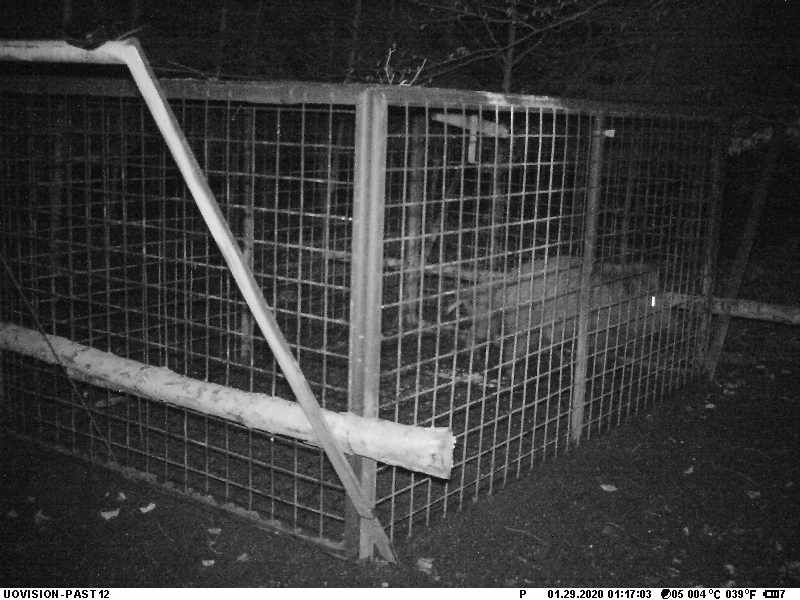

Supplement: Supplementary file 1 — Supplementary Information. [file 41598_2021_95682_MOESM1_ESM.zip › SEM_photos complete/PIC0762.jpg]

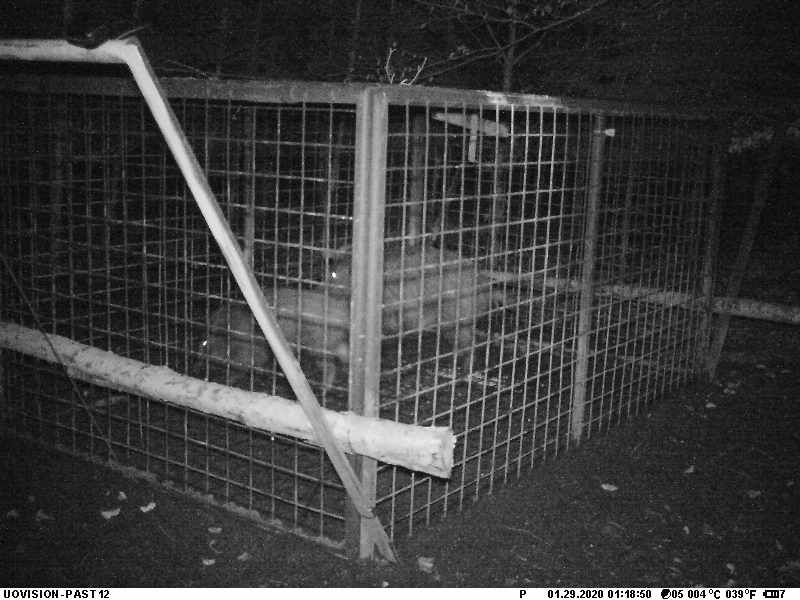

Supplement: Supplementary file 1 — Supplementary Information. [file 41598_2021_95682_MOESM1_ESM.zip › SEM_photos complete/PIC0763 (1).jpg]

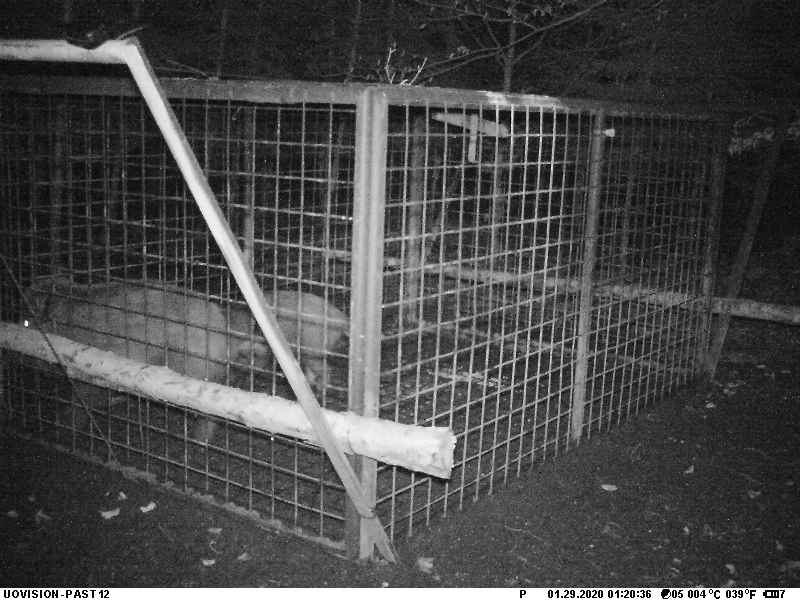

Supplement: Supplementary file 1 — Supplementary Information. [file 41598_2021_95682_MOESM1_ESM.zip › SEM_photos complete/PIC0764 (1).jpg]

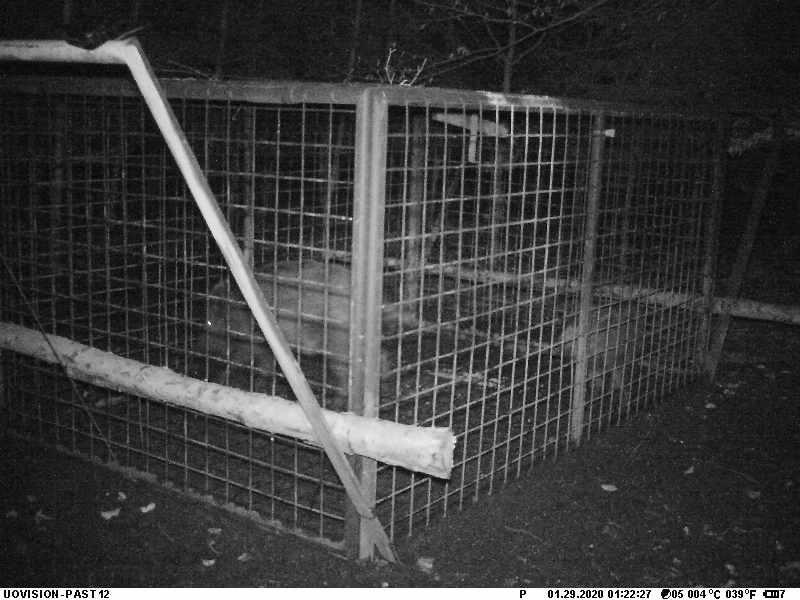

Supplement: Supplementary file 1 — Supplementary Information. [file 41598_2021_95682_MOESM1_ESM.zip › SEM_photos complete/PIC0765 (1).jpg]

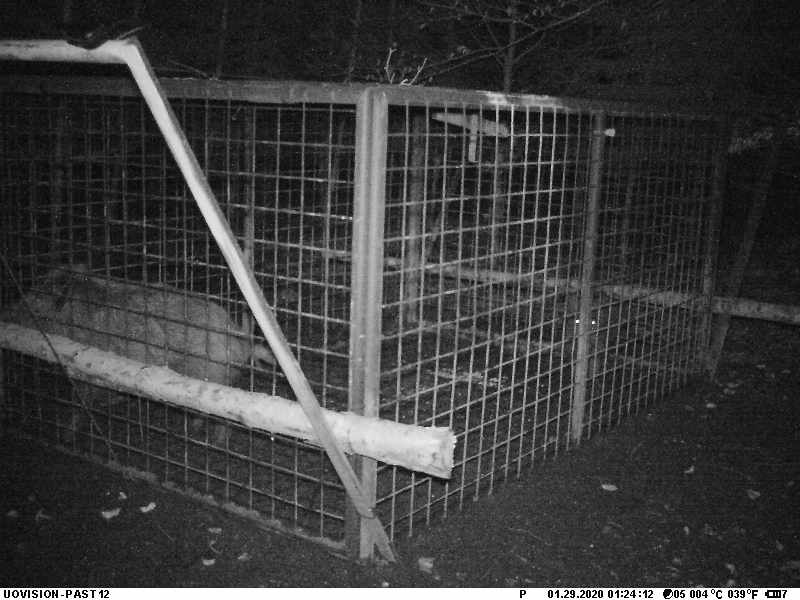

Supplement: Supplementary file 1 — Supplementary Information. [file 41598_2021_95682_MOESM1_ESM.zip › SEM_photos complete/PIC0766.jpg]

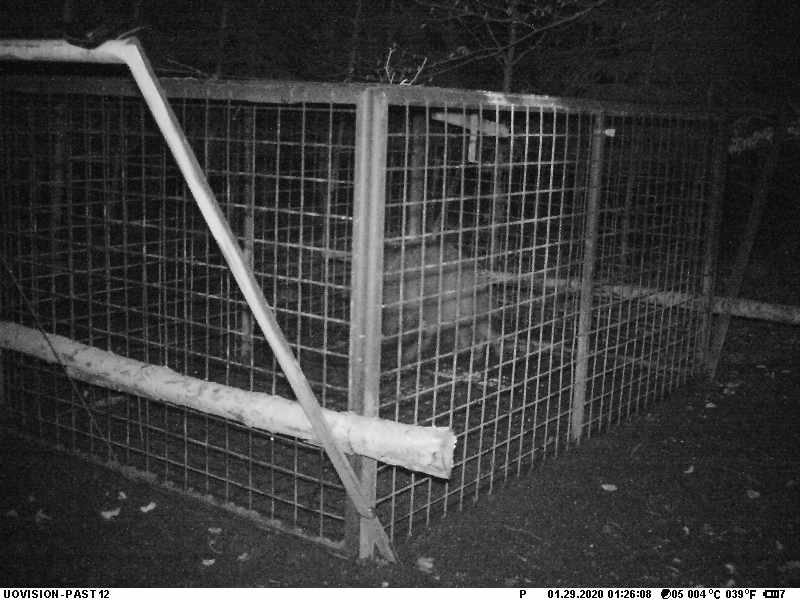

Supplement: Supplementary file 1 — Supplementary Information. [file 41598_2021_95682_MOESM1_ESM.zip › SEM_photos complete/PIC0767 (1).jpg]

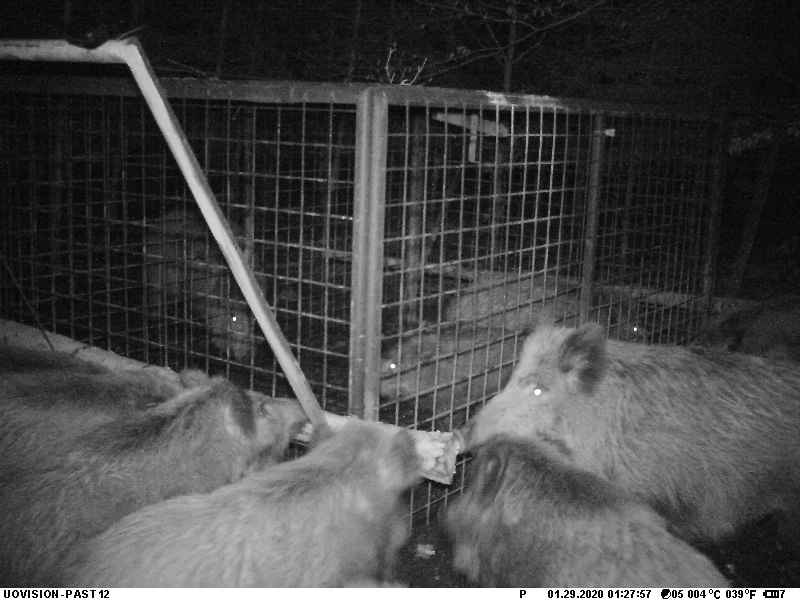

Supplement: Supplementary file 1 — Supplementary Information. [file 41598_2021_95682_MOESM1_ESM.zip › SEM_photos complete/PIC0768 (1).jpg]

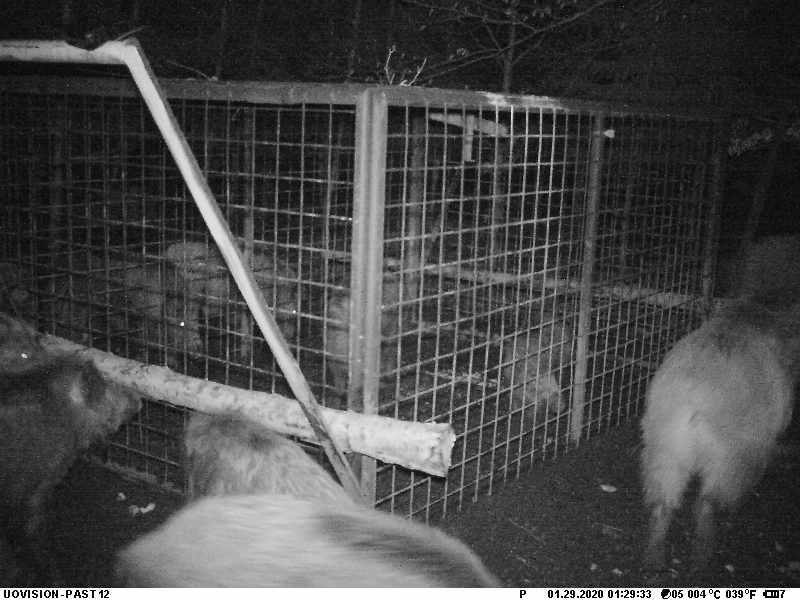

Supplement: Supplementary file 1 — Supplementary Information. [file 41598_2021_95682_MOESM1_ESM.zip › SEM_photos complete/PIC0769 (1).jpg]

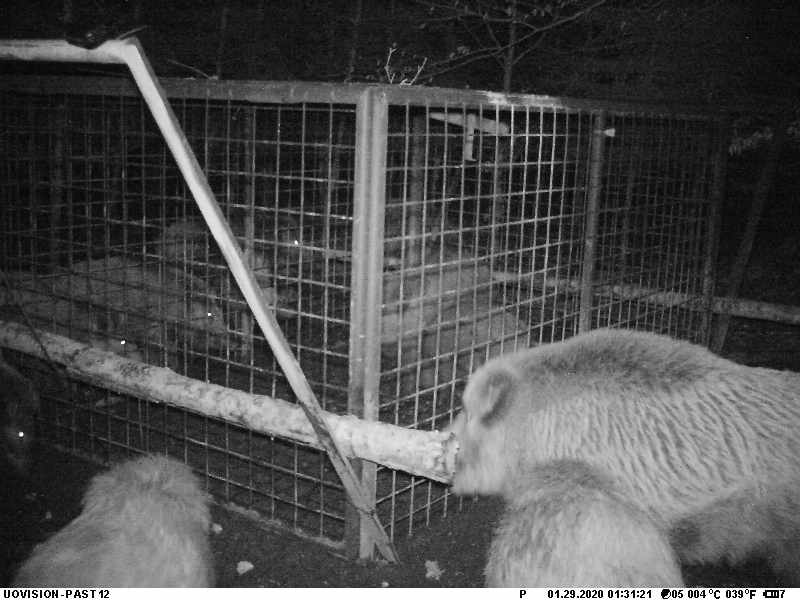

Supplement: Supplementary file 1 — Supplementary Information. [file 41598_2021_95682_MOESM1_ESM.zip › SEM_photos complete/PIC0770 (1).jpg]

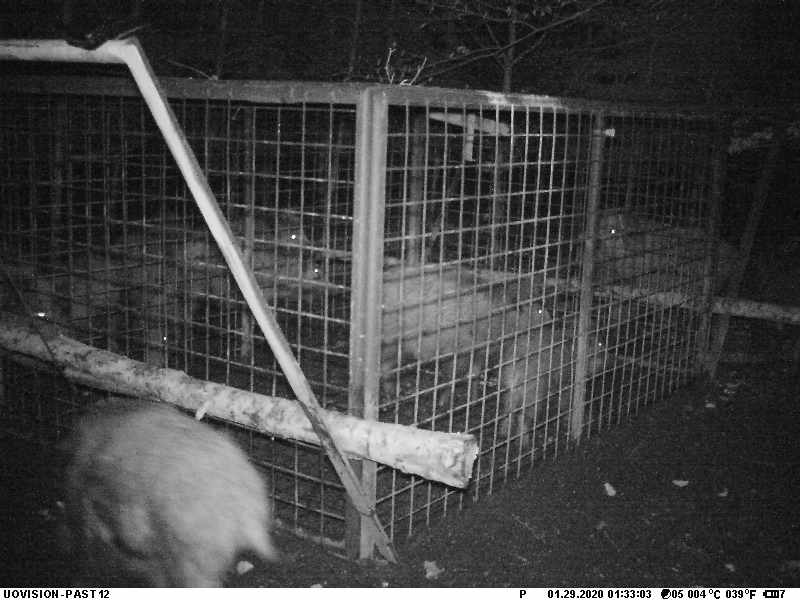

Supplement: Supplementary file 1 — Supplementary Information. [file 41598_2021_95682_MOESM1_ESM.zip › SEM_photos complete/PIC0771 (1).jpg]

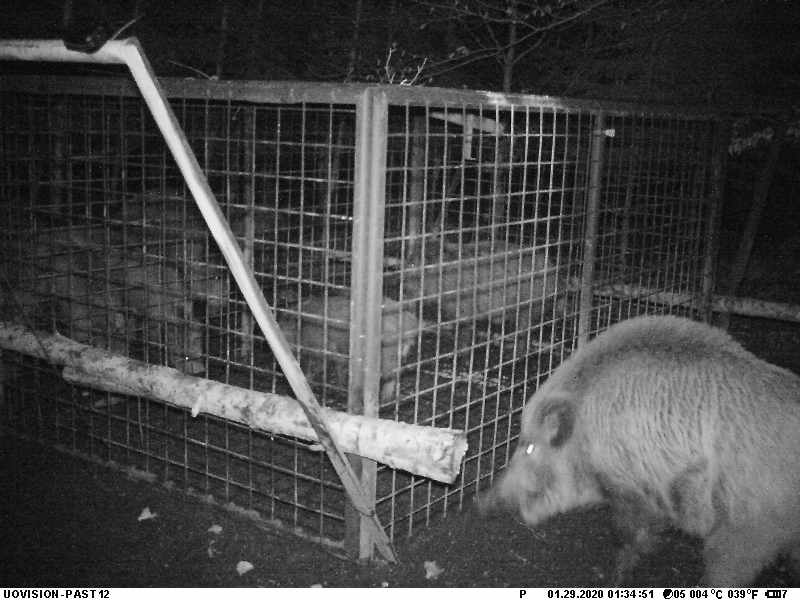

Supplement: Supplementary file 1 — Supplementary Information. [file 41598_2021_95682_MOESM1_ESM.zip › SEM_photos complete/PIC0772 (1).jpg]

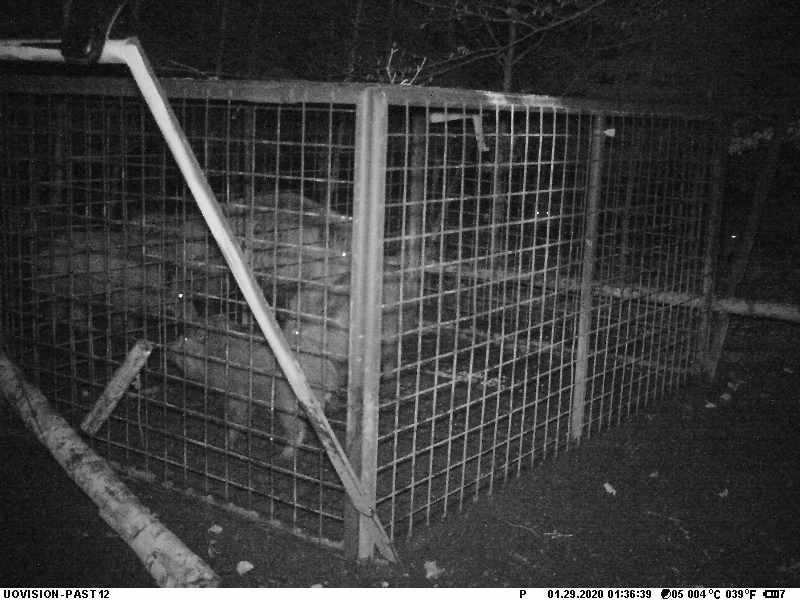

Supplement: Supplementary file 1 — Supplementary Information. [file 41598_2021_95682_MOESM1_ESM.zip › SEM_photos complete/PIC0773 (1).jpg]

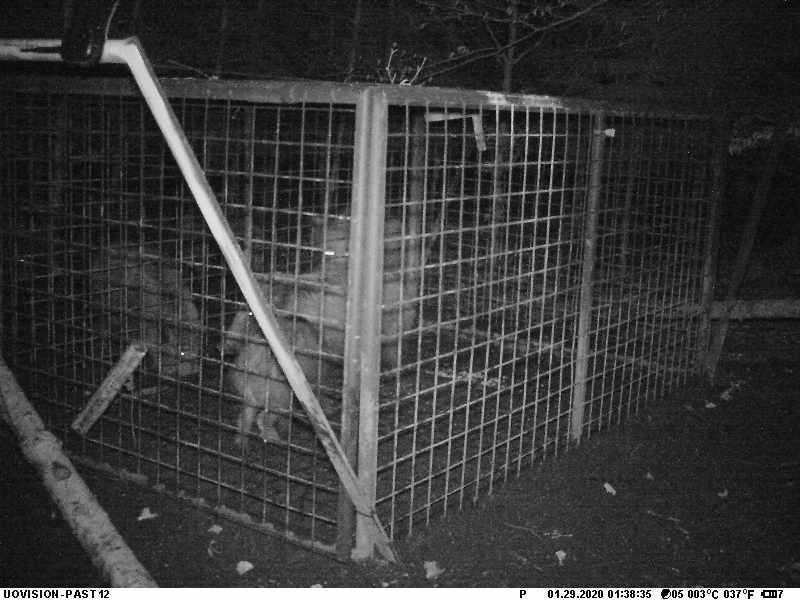

Supplement: Supplementary file 1 — Supplementary Information. [file 41598_2021_95682_MOESM1_ESM.zip › SEM_photos complete/PIC0774 (1).jpg]

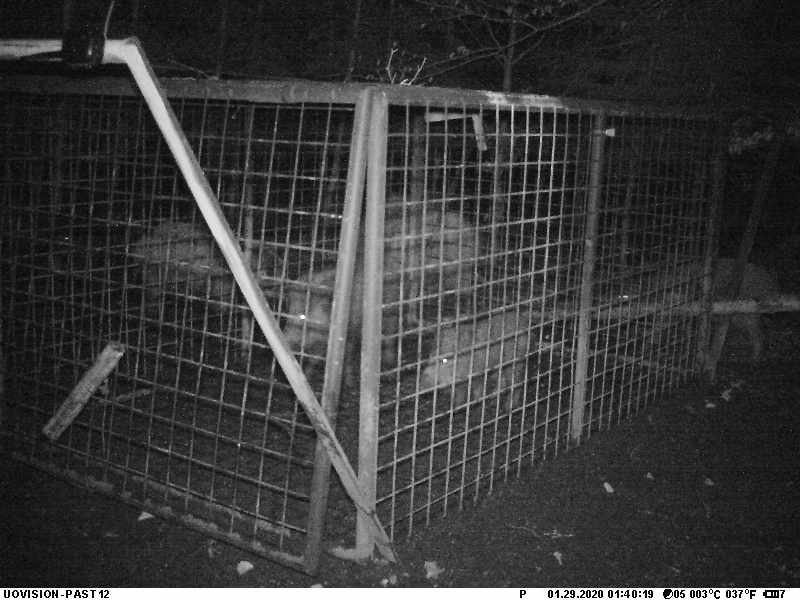

Supplement: Supplementary file 1 — Supplementary Information. [file 41598_2021_95682_MOESM1_ESM.zip › SEM_photos complete/PIC0775 (1).jpg]

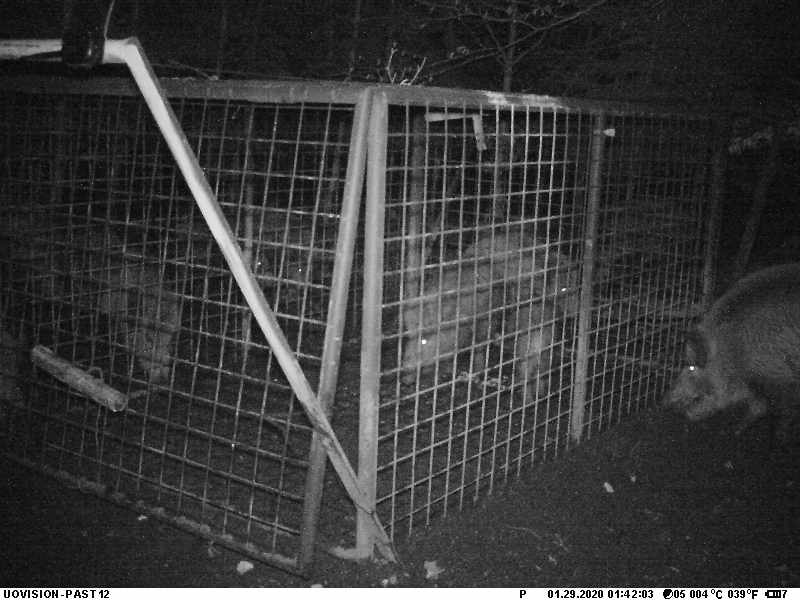

Supplement: Supplementary file 1 — Supplementary Information. [file 41598_2021_95682_MOESM1_ESM.zip › SEM_photos complete/PIC0776 (1).jpg]

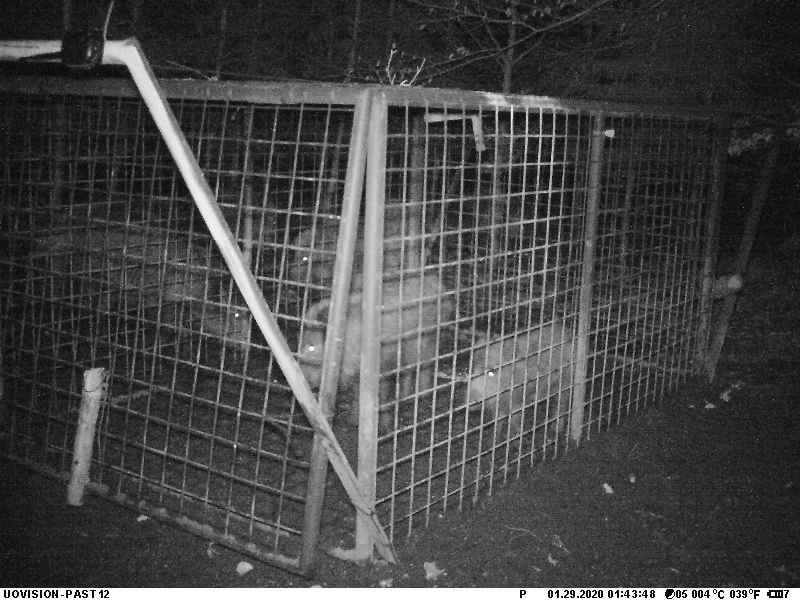

Supplement: Supplementary file 1 — Supplementary Information. [file 41598_2021_95682_MOESM1_ESM.zip › SEM_photos complete/PIC0777 (1).jpg]

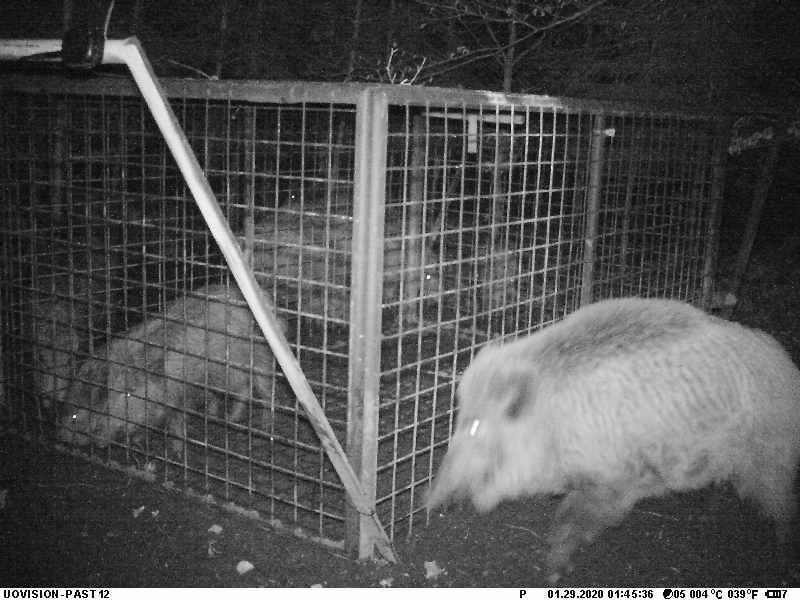

Supplement: Supplementary file 1 — Supplementary Information. [file 41598_2021_95682_MOESM1_ESM.zip › SEM_photos complete/PIC0778 (1).jpg]

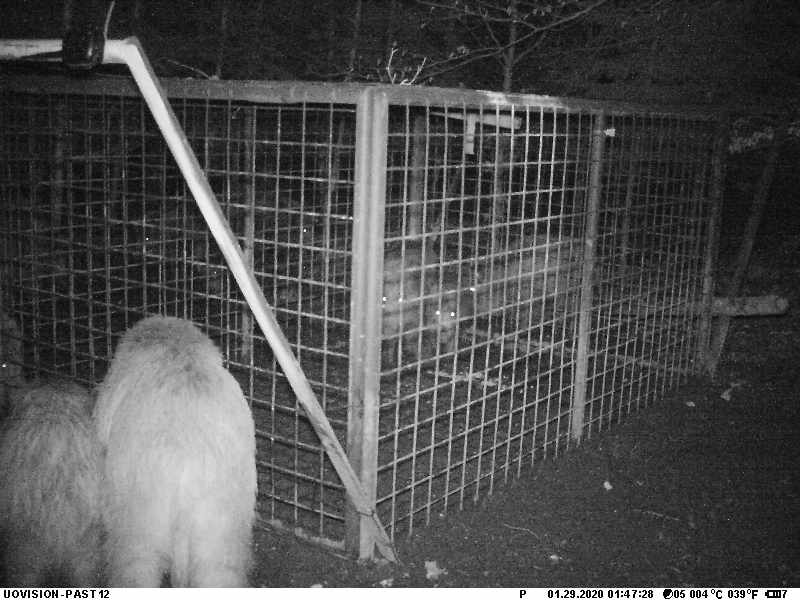

Supplement: Supplementary file 1 — Supplementary Information. [file 41598_2021_95682_MOESM1_ESM.zip › SEM_photos complete/PIC0779 (1).jpg]

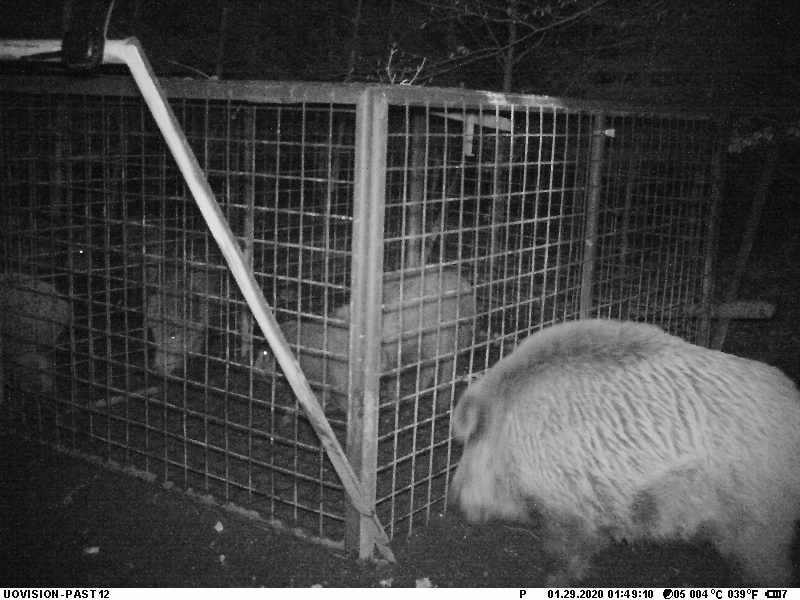

Supplement: Supplementary file 1 — Supplementary Information. [file 41598_2021_95682_MOESM1_ESM.zip › SEM_photos complete/PIC0780 (1).jpg]

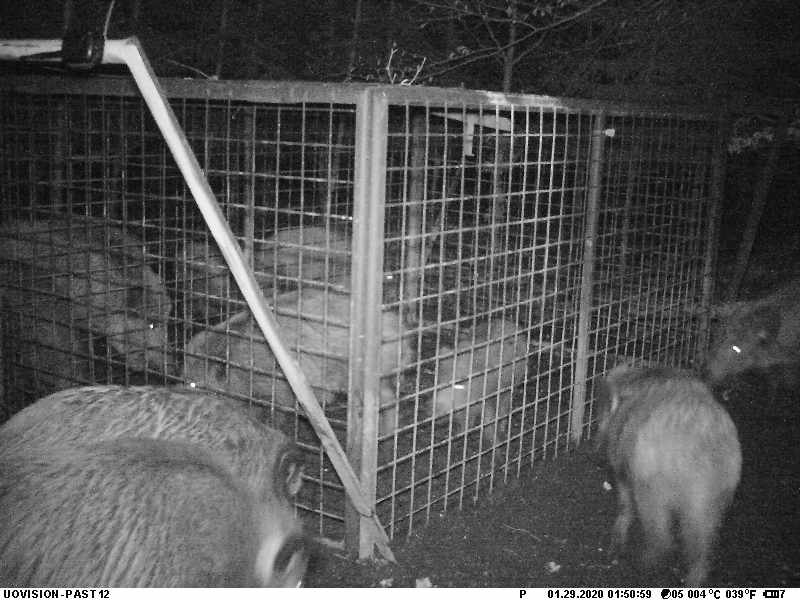

Supplement: Supplementary file 1 — Supplementary Information. [file 41598_2021_95682_MOESM1_ESM.zip › SEM_photos complete/PIC0781 (1).jpg]

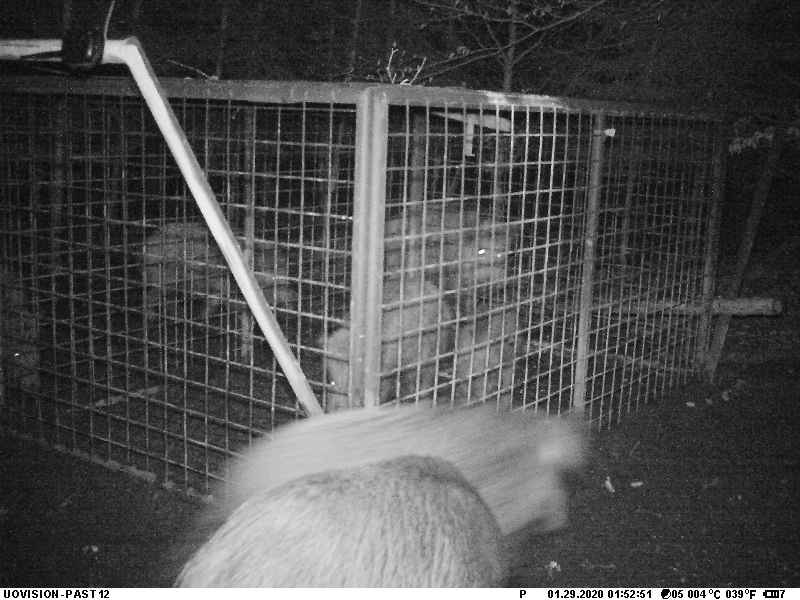

Supplement: Supplementary file 1 — Supplementary Information. [file 41598_2021_95682_MOESM1_ESM.zip › SEM_photos complete/PIC0782 (1).jpg]

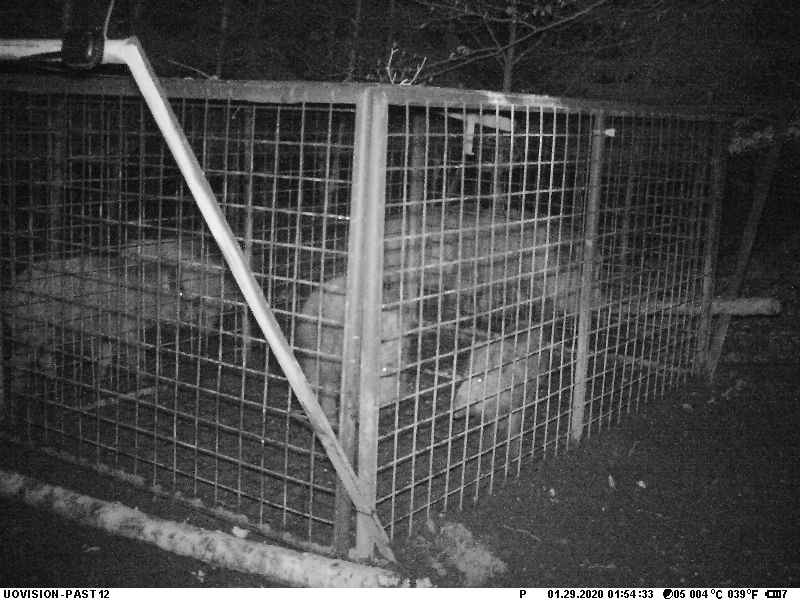

Supplement: Supplementary file 1 — Supplementary Information. [file 41598_2021_95682_MOESM1_ESM.zip › SEM_photos complete/PIC0783 (1).jpg]

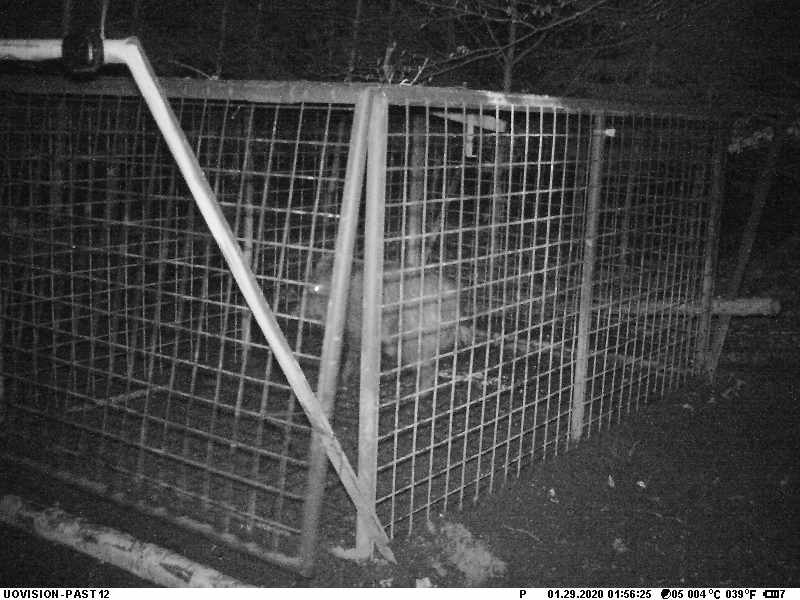

Supplement: Supplementary file 1 — Supplementary Information. [file 41598_2021_95682_MOESM1_ESM.zip › SEM_photos complete/PIC0784 (1).jpg]

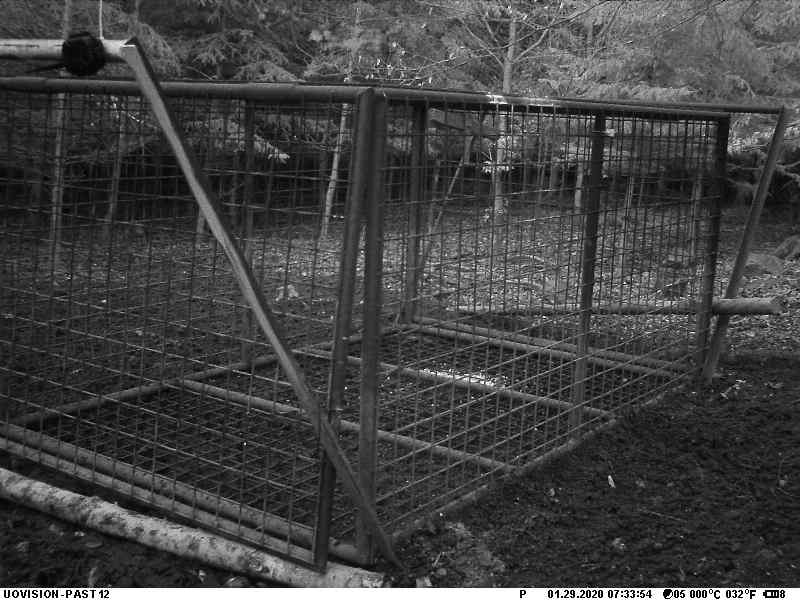

Supplement: Supplementary file 1 — Supplementary Information. [file 41598_2021_95682_MOESM1_ESM.zip › SEM_photos complete/PIC0785 (1).jpg]

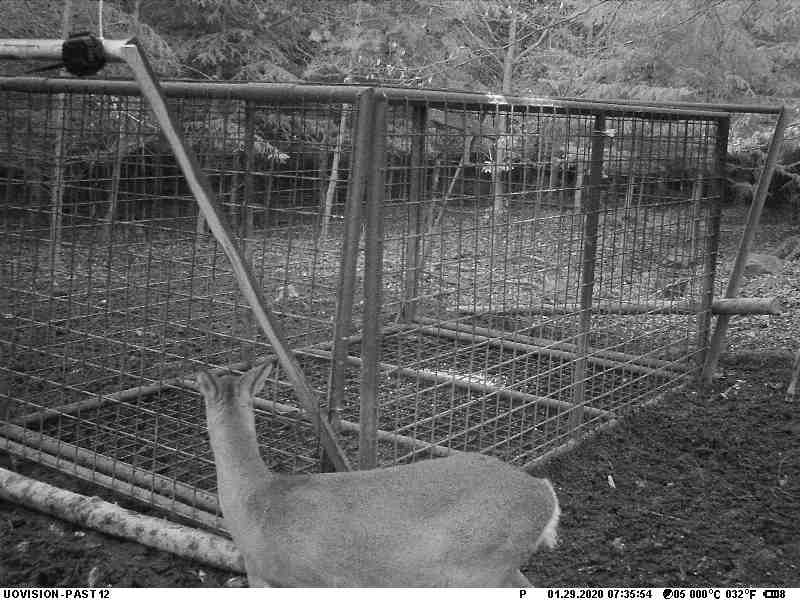

Supplement: Supplementary file 1 — Supplementary Information. [file 41598_2021_95682_MOESM1_ESM.zip › SEM_photos complete/PIC0786.jpg]
